# Present and Future of the White‐Tailed Laurel Pigeon (*Columba junoniae*) on Gran Canaria Island

**DOI:** 10.1002/ece3.71580

**Published:** 2025-06-21

**Authors:** Gonzalo Albaladejo‐Robles, José Manuel Caballero Fernández, Yarci Acosta Santana

**Affiliations:** ^1^ Research Department Natural History Museum London UK; ^2^ Centre for Biodiversity and Environment Research, Department of Genetics, Evolution and Environment University College London London UK; ^3^ Independent Scholar; ^4^ Sociedad Española de Ornitología (SEO/BirdLife), delegación de Canarias La Laguna Canary Islands Spain

**Keywords:** birds, climate change, conservation, conservation evaluation, islands, management, reintroduction

## Abstract

Due to their evolutionary history and restricted distribution islands species are particularly vulnerable to human impacts and extinction. Consequently, many islands' species have been extirpated, causing complete or local extinctions. Reintroductions are useful, although challenging, tools to restore ecosystems and halt biodiversity loss. In this work, we evaluate the reintroduction success of the endemic white‐tailed laurel pigeon ( *Columba junoniae* ) on the island of Gran Canaria. We also explore its future potential distribution under different scenarios of climate change in the Canary Islands (Spain). We used a combination of Maximum Entropy models (MaxEnt), trained with spatial records within the whole range of the species, to model the potential distribution of *C. junoniae* on the island of Gran Canaria, where it was recently reintroduced. We compared this potential distribution with the actual distribution of the species in the reintroduction area. Furthermore, we used multiple scenarios of climate change to analyze the likely changes in the species' suitable habitat. We found that *C. junoniae* has colonized most of its potential habitat in the new reintroduction area. Overall, this marks that the reintroduction has successfully facilitated the spread and settlement of the species. However, our analysis also showed that this habitat is expected to suffer future fragmentations and contractions under different climate change scenarios. Based on our research, *C. junoniae* has colonised most of its potential habitat within its new distribution area. Although this is a huge milestone for the conservation of the species, future changes might jeopardise the species' future. In this scenario of accelerated environmental change, microhabitats and niche refuges can alleviate this situation. Our results also suggest that restoration of native forests is fundamental to ensure the species' long‐term persistence and ecosystems' resilience against climate and land‐use changes. This work sets the principles for the evaluation of the reintroduction of *C. junoniae* in Gran Canaria, as well as the long‐term conservation strategy for the species in its new distribution area.

## Graphical abstract

Reintroduction projects are fundamental for the restoration of ecosystems and biodiversity conservation. In this work, we evaluate the success of the reintroduction of the white‐tailed laurel pigeon on Gran Canaria island. We found that the species has colonized most of its potential habitat in the new distribution area but that such niche is likely to be reduced in the mid‐to long‐ term future due to climate change. We propose climate change mitigation strategies to be planned and implemented in the early stages of any reintroduction project.

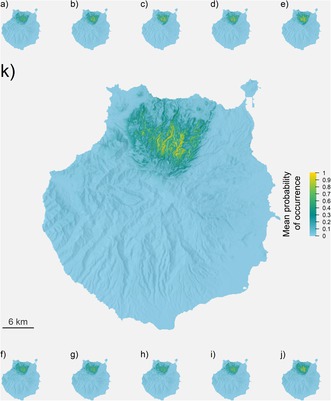

## 1 Introduction

Despite representing roughly 7% of the emerged land surfaces on the planet, islands contribute disproportionately to global biodiversity, hosting approximately 20% of the world's biota (Veron et al. 2019; Fernández‐Palacios et al. 2021). Due to their isolation and unique evolutionary history, island ecosystems are usually more vulnerable to human‐driven environmental changes and prone to extinctions (Fernández‐Palacios et al. 2021). Of all known recorded species extinctions, approximately 75% have happened on islands (Fernández‐Palacios et al. 2021; Sayol et al. 2024) with a higher proportion of them affecting bird species (see Matthews et al. 2024). In the actual context of vulnerability and accelerated biodiversity loss (Ceballos et al. 2017; Ceballos and Ehrlich 2023), conservation strategies such as translocations or reintroductions are powerful tools for species conservation, habitat restoration, and ecosystem services preservation (e.g., Soorae 2018). However, reintroductions are costly, challenging, and hard to maintain long‐term (e.g., Soorae 2018; Morris et al. 2021). These factors make long‐term planning and evaluation of these projects challenging (Seddon et al. 2007). Here we focus on the outcomes, challenges, and future conservation implications of the white‐tailed laurel pigeon ( *Columba junoniae* ) reintroduction on Gran Canaria Island (Canary Islands, Spain).

**FIGURE 1:**
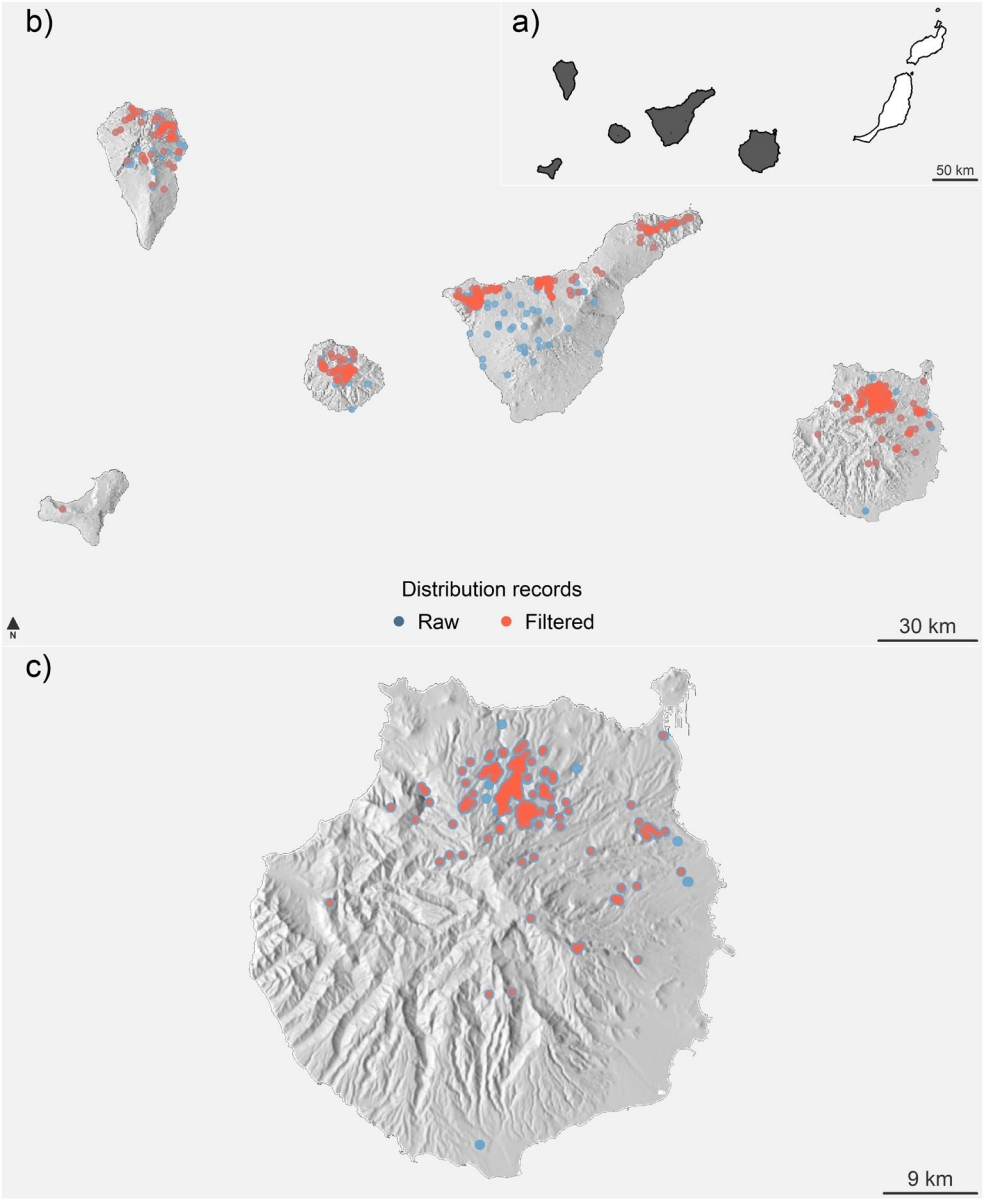
Distribution of *C. junoniae* in the Canary Islands and Gran Canaria. (a) Dark polygons indicate the distribution of *C. junoniae* across the Canary Islands. (b) Blue dots represent all compiled observations from GBIF and Life+Rabiche, with red dots marking the subset used for the final analysis. (c) Detailed map of *C. junoniae* observations on Gran Canaria, highlighting data density. A numerical summary of records by island can be seen in Table S2. Shaded maps depict the topographic profiles of the islands, derived from a high‐resolution digital elevation model (DEM). Polygons and elevation data were sourced from the open repositories of the Instituto Geográfico Nacional (https://www.ign.es/).

*C. junoniae* is one of the two endemic pigeons that inhabit the Canarian archipelago. Its natural range is ascribed to the westernmost islands of this archipelago: La Gomera, El Hierro, La Palma, and Tenerife (Figure 1a,b); (Martín et al. 2000). However, the previous presence of this species in Gran Canaria Island cannot be ruled out. In the past, Gran Canaria possessed mature laurel and thermophilous forests, the preferred habitat for *C. junoniae* . Furthermore, the decimation of such forests favoring urban development and plantations coincides with the latest historical reference of endemic pigeons ( *Columba bollii* ) on the island, around 1870 (Martín et al. 2013). Additionally, fossils from endemic pigeons have been found on the island (Florit, 1989). Although it is clear that these remains belong to one or both of the endemic pigeons, a final identification is still needed (Martín et al. 2013). Such circumstances, along with the classification of *C. junoniae* as vulnerable by the Spanish National List of Species Under Special Protection (Real Decreto 139/2011, n.d.), made this species a suitable candidate for reintroduction under the IUCN Species Survival Commission guidelines (IUCN/SSC 2013), and a perfect banner for the restoration of the laurel and thermophilous forests of Gran Canaria. This way, since the early 2000's a series of studies, reports, and captivity breeding experiments set the basis for the reintroduction project of the white‐tailed laurel pigeon in Gran Canaria (Life+ Rabiche NAT/ES/000354; Martín et al. 2000).

Reintroduction projects are complex and multidisciplinary endeavours (IUCN/SSC 2013); the Life+ Rabiche was no different. After 13 years of preparation and nine of execution, the project ended in 2023 after achieving several milestones for species conservation. As a result of this project, between 2012 and 2023, more than 388 captive‐bred pigeons were liberated in Gran Canaria, with born‐free individuals recorded since 2013. Additionally, approximately 220 new hectares of laurel and thermophilous forest were planted, restored, or protected thanks to the initiatives inspired by the project and the collaboration of the private and public sectors (Martín et al. 2020). Despite this, it is still unknown if future planning and management are needed to secure the success of the species.

Here, we evaluate the success of the white‐tailed laurel pigeon reintroduction and its future on the island of Gran Canaria by evaluating the likely change of its distribution under different scenarios of climate change. Understanding the factors driving species distributions is fundamental when designing and evaluating any conservation project (Armstrong et al. 2013; Richardson and Whittaker 2010). This is especially important when we consider reintroduction projects. Species Distribution Models (SDMs) are statistical models designed to reconstruct and project species niches using species spatial records and their associated environmental factors (Peterson et al. 2011; Pearce 2012; Holt 2009; Vandermeer 1972). To model the potential distribution of *C. junoniae* in Gran Canaria island, we compiled distribution records of the species from all the available sources, along with a combination of remote sensing environmental data. This included climatic, topographic and land use data as well as a combination of 15 different socio‐economic and climatic scenarios (Riahi et al. 2017). This information was then combined into several Maximum Entropy models (MaxEnt), which were combined and averaged to define the niche of *C. junoniae* in the reintroduction area. By doing this, we aim to address the following questions: (1) Has the white‐tailed laurel pigeon reached or occupied its potential habitat on the island of Gran Canaria? (2) How is the potential distribution of the species going to change in the future (2071–2100)? (3) Which conservation actions can we implement to attenuate the effects of climate change on the species?

## 2 Materials and Methods

### 2.1 Presence Data

The white‐tailed laurel pigeon is endemic to the Canarian archipelago and is distributed across the western islands: La Palma, La Gomera, El Hierro, Tenerife, and since 2012 Gran Canaria where it was reintroduced (Figure 1a,b) (Martín et al. 2020). To evaluate if *C. junoniae* has reached its potential distribution on the island of Gran Canaria, we needed to compare the actual distribution of the reintroduced individuals with the potential distribution of the species on the island (e.g., Smeraldo et al. 2017). To do so, we combined two different datasets; one contained all the presence records recorded during the monitoring of the Life+ Rabiche, and a second one contained all the available records of the species from the rest of the Canary Islands.

The presence data of *C. junoniae* in Gran Canaria Island was obtained directly from the monitoring database of the Life+ Rabiche. This database contains more than 9000 georeferenced presence records from 2013 to 2023, which were recorded using different methods (observation points, transects, radio‐tracking and GPS‐tracking). This data covers an area of approximately 30 km^2^ on the island of Gran Canaria (Figure 1c). However, the species also inhabits the western islands of the archipelago. To obtain presence data for the rest of its range, we retrieved all the available records from the Global Biodiversity Information Facility (https://www.gbif.org) (GBIF hereafter). We retrieved all the available information for the species from 1970 to 2023. GBIF data combines citizen science, official monitoring data from governments and NGOs, individuals, or user observations, and scientific collections. All presence records, GBIF and Life+ Rabiche, were filtered to remove spatial bias and erroneous records (e.g., Supporting Information—S1 and Table S1). After this filtering, we reduced the initial dataset to 633 records (307 from the Life+ Rabiche and 326 from GBIF).

Once filtered, the Life+ Rabiche and GBIF records were combined into a single dataset, and a Mantel test was performed to determine if the remaining points present spatial autocorrelation at any given distance (Mantel 1967). This test evaluates the correlations between the distance matrix formed by our presence points and the values of the environmental variables (see 2.2 Environmental variables). If high correlations are detected (values closer to one) it is assumed that observation distances are directly related to the environmental gradients, and therefore there is spatial autocorrelation. In these cases, further spatial thinning or data filtering is needed. In our case, the Mantel test revealed weak, lower than 0.15, but statistically significant (*p* < 0.001) spatial autocorrelations at distances from 50 to 1400 m (Figure S1a). Since the spatial autocorrelations over very short spatial distances were low, we did not apply further spatial thinning to the presence records.

### 2.2 Environmental Variables

#### 2.2.1 Climatic Data

We selected a combination of climatic, land cover/use, and topographic variables. In the case of the climatic variables, we used the high‐resolution CanaryClim version 1.0 (Patiño et al. 2023) (CanaryClim—Climatic maps (figshare.com)). This dataset contains 19 different climatic variables relevant to the species distribution. This dataset follows the same structure and format as Bioclim (Karger et al. 2017) (https://chelsa‐climate.org/bioclim/) which is widely used in species distribution modelling and ecology (e.g., Booth 2018). The variables present in CanaryClim had a spatial grid resolution of 100 m; this makes this dataset ideal for the modelling of species in small areas (Seo et al. 2009) and has been used to model the present and future potential habitat of species in the Canary Islands (Patiño et al. 2023). The spatial resolution at which environmental variables are recorded can have a huge impact on the characterisation of the habitat and subsequently on the results of any SDM (Farashi and Alizadeh‐Noughani 2018). We consider that a resolution of 100 m allows us a good representation of the complex terrain of the Canary Islands (Patiño et al. 2023). Furthermore, from a landscape‐scale point of view, we consider that this resolution is relevant to describe the potential habitat of *C. junoniae* . Additionally, CanaryClim is based on climatic data gathered locally across meteorological stations across the Canarian archipelago (Patiño et al. 2023). For the modelling of the potential distribution of *C. junoniae*, we selected four present and future climatic variables: isothermality (bio_03), temperature seasonality (bio_04), precipitation of the driest month (bio_14), and precipitation seasonality (bio_15). This selection was based on their biological potential as well as on the auto‐correlation and variable inflation factor of the different combinations of variables (e.g., Supporting Information—S3 and Tables S3, S4).

On top of the present climatic variables (CanaryClim, same as Bioclim, use average values from 1979 to 2013), this dataset also contains future climatic data for three different shared socioeconomic pathways (SSP) scenarios: SSP 1–2.8, SSP 3–70, and SSP 5–8.5 (Riahi et al. 2017) for the 2070–2100 period. These scenarios provide estimates of human CO_2_ emissions under different developmental and biodiversity conservation strategies. The climatic data for the future SSP scenarios are projected using five different global atmospheric circulation models (IPSL, GFDL, MPI, MRI and UKESM1) with distinct sensitivities to CO_2_ increments (Riahi et al. 2017). Therefore, there is not a unique future climatic scenario but a combination of projections with different sensitivities and outcomes. For this reason, we used the combination of the SSP scenarios and atmospheric models to evaluate the future distribution of *C. junoniae* in Gran Canaria. In total, we used 15 different future climatic scenarios.

#### 2.2.2 Land Use Information

The land use/cover information was retrieved from the European Space Agency Climate Change Initiative Land Cover (ESA CCI LCP, http://www.esa‐landcover‐cci.org) version 2.0.7 (ESA‐LC hereafter). This database contains time series of high‐resolution global land‐cover information from 1991 to 2020 at a grid resolution of 300 m (Harper et al. 2023). To coordinate as much as possible the land‐cover information with our monitoring information and climatic variables, we selected the 2015 land‐cover data (most of the monitoring data ranged from 2014 to 2016). The ESA‐LC dataset contains 36 different categories of land cover. Most species distribution models do not scale well with categorical data (Phillips et al. 2006). Therefore, we transformed these categorical variables into percentages of land cover for six plant functional types compatible with the ESA‐LC information (Li et al. 2017); tree, shrub, cropland, grass, urban and bare soil. The final plant functional types reflect the percentage of total cover, between 0 and 1, of that plant type for a given pixel. From this pool of variables, we selected tree, shrub, crop and grass cover (e.g., Supporting Information—S1).

We adapted the resolution of the land‐cover information from 300 to 100 m to make it compatible with the climatic data and to better reflect the spatial complexity of the Canary Islands. We achieve this downscaling by using bilinear interpolation (e.g., Latombe et al. 2018).

#### 2.2.3 Topographic Information

In addition to the land‐cover information, we collected four topographic variables to summarize the topographic landscape complexity found on the island of Gran Canaria: elevation, roughness, aspect, and slope. Elevation is an important factor, delimiting the main vegetation communities across the Canarian archipelago. Similarly, roughness, aspect, and slope are important factors when describing the complexity of ravines, canyons, and other formations found on Gran Canaria Island (Figure 1c). These variables were derived and calculated from a 25 m grid resolution digital elevation model (DEM) obtained from the Instituto Geográfico Nacional (http://centrodedescargas.cnig.es/CentroDescargas/index.jsp). To keep consistency with the rest of the environmental information, we upscaled this topographic information to 100 m using bilinear interpolation (e.g., Chakraborty et al. 2020) (more details on variable calculation and transformation in Supporting Information—S1).

### 2.3 Species Distribution Modelling

To model the potential distribution of *C. junoniae* in Gran Canaria, we used data spanning the species' known distribution across the Canarian archipelago. This ensured a more accurate description of the species' niche and, therefore, a more reliable estimation of its potential distribution in Gran Canaria. To do this, we combined the distribution data and the environmental information into a maximum entropy modelling algorithm (MaxEnt) (Phillips et al. 2006). MaxEnt is a presence‐only species distribution modelling algorithm that uses species presence information, alongside spatial environmental information, to reconstruct and project their niche (Phillips et al. 2006). This method shows a series of advantages when compared to other SDM approaches: (1) It is a semi‐unsupervised machine learning algorithm, which makes it easy to implement and able to work with scarce data; (2) is a well‐tested method, extensively used in ecology and conservation, and able to produce more accurate results than other SDM methods (Fitzgibbon et al. 2022); (3) it can work with highly multidimensional data; (4) it offers access to base parametrisation and optimisation, which make it flexible and customisable (Phillips et al. 2006). All this makes it ideal for the aims of our study.

**FIGURE 2:**
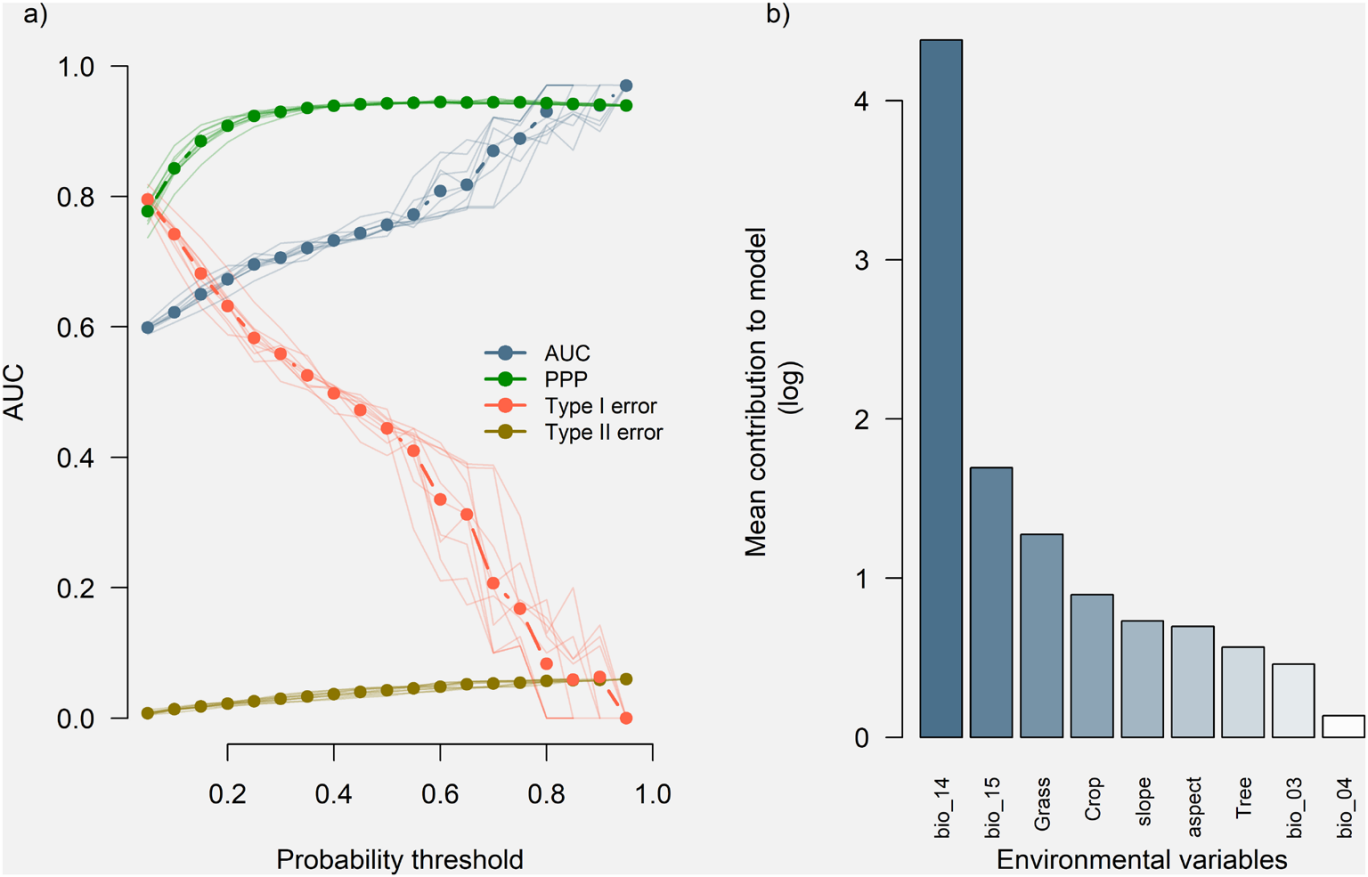
Performance of MaxEnt models and contribution of environmental variables. (a) Model performance: Lines represent the performance of the 10 final selected models at different probability thresholds, evaluated using four accuracy metrics: AUC (area under the curve), PPP (proportion of positive predictions), and Type I & II errors. Dashed lines and points indicate the average values for each metric. (b) Environmental variable contributions: Bars show the average contribution of each variable to the models. Variables include bio_04 (temperature seasonality), bio_03 (isothermality), Tree (percentage of tree cover), terrain aspect and slope, Crop (percentage of crop cover), Grass (percentage of grassland cover), bio_15 (precipitation seasonality) and bio_14 (precipitation of the driest month). Precipitation seasonality and precipitation of the driest month are the most influential, explaining over 85% of model variance.

For the distribution models of *C. junoniae* we used 633 presence points covering the whole range of the species (307 observations from the Life+ Rabiche monitoring program and 326 from GBIF). These presence points were divided into train (506 presence points) and test (127 presence points) groups and 10.000 randomly sampled spatial records across the species range (e.g., Supporting Information—S2 and Figure S2). These background points were also split into train and test data (8000 and 2000 records respectively). Models were fitted using a set of nine environmental variables (four climatic variables, three land cover variables, and two topographic variables) that were previously selected: seasonal temperature and precipitation, precipitation of the driest month, percentage of herbaceous and tree cover, percentage of crop coverage, aspect, and slope (Figure 2b) (variable selection details in Supporting Information—S3).

MaxEnt algorithm was randomly clamped to generate a wider diversity of responses (Redding et al. 2017), we ran a total of 100 different distribution models. From this pool of models, we measured their performance at different probability thresholds (Figure 2a) and selected the 10 models that showed a higher average Area Under the Curve (AUC hereafter). The AUC is a validation metric that measures how accurately a model discriminates between classes when running a classification (Hanley and McNeil 1982). The AUC returns a value within the range of 0–1, where 0 is perfectly incorrect discrimination, 0.5 corresponds to the performance of a random classifier, and 1 corresponds to a perfect classification. In our case, values of AUC closer to 1 denote a model with a higher discrimination capacity between presences and absences.

Once models were selected, their predictions over the island of Gran Canaria were processed and the cumulative arithmetic mean was calculated. This mean represents the potential distribution of *C. junoniae* on the island of Gran Canaria in a probability gradient from 0 to 1, where values close to cero represent a low probability of habitat suitability and values closer to one represent a high probability of habitat suitability for the species. To calculate the area of maximum probability of occurrence (AMPO hereafter) for the species, we used the probability threshold at which the Kappa parameter was the highest (e.g., Zhang et al. 2015). The Kappa parameter is a metric that measures the level of congruency between two dependent categories, in our case presence records (1) and background points (0). The values of Kappa range from cero (without congruency) to one (perfect congruency). This parameter was calculated using the testing data across the full extent of the model. Once the probability threshold of each model was fixed, we classified their predictions into presence (1) and absence (0) and used the first to delimit the AMPO of *C. junoniae* . This AMPO area is now a discrete surface, rather than a continuous estimation of probability of occurrence. The occupancy area for the individual models was then spatially combined into a single compound area of occupancy or global AMPO.

Since our area of interest is Gran Canaria, we focus on the occupancy areas of this island. These models were then compared with current distribution records of the species in the reintroduced area to assess whether the species has reached all its potential habitats, identify areas for monitoring or population reinforcement, and potential limitations for the expansion of the species in Gran Canaria.

Using the selected models, we predicted the future potential distribution of *C. junoniae* using the climatic data for the different socioeconomic and atmospheric scenarios while keeping the land use and topographic data constant. Model predictions were averaged across socioeconomic scenarios, and the most probable area of future species occupancy was again calculated using the probability threshold previously calculated for each model; this is the point at which the Kappa parameter was maximum. Same as before, occupancy areas were combined on a socioeconomic scenario and atmospheric model basis.

Details about model adjustment, variable selection and model fit can be found in the Supporting Information—S3, Tables S3, S4.

## 3 Results

### 3.1 Model Performance and Selection

The 10 best‐fitted MaxEnt models scored AUC values above or equal to 0.75. Other supplementary metrics such as the type I and II errors and the Probability of Positive Prediction (PPP) showed that models were effective and precise when it came to correctly classifying presence and absence records (Figure 2a). Although models were adjusted and evaluated using the whole Canarian archipelago, we focused only on the results regarding Gran Canaria Island, where *C. junoniae* has been reintroduced.

### 3.2 Distribution of *C. junoniae* in Its Reintroduction Area

**FIGURE 3:**
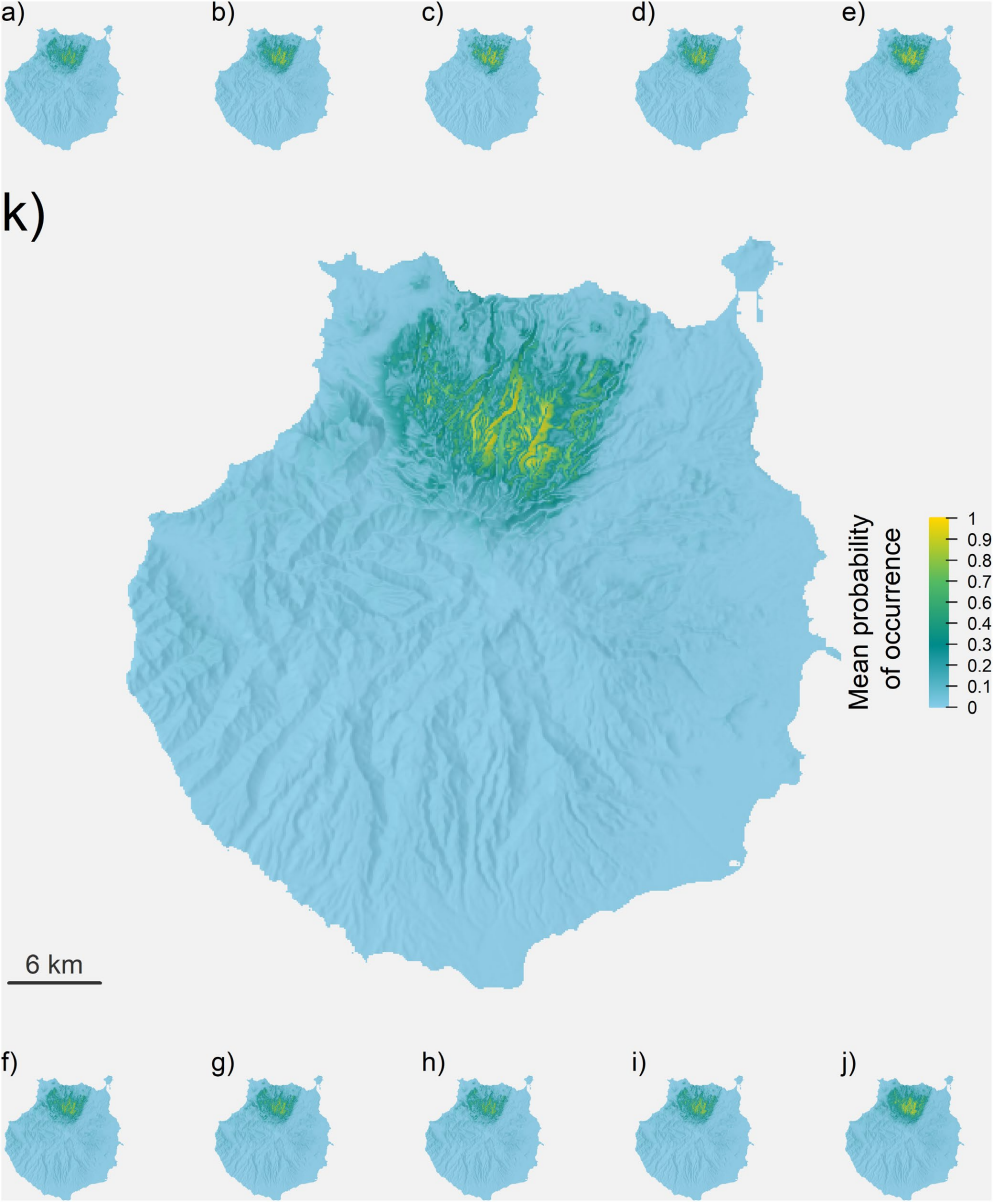
MaxEnt models used for the calculation of the present distribution of *C. junoniae* on the Island of Gran Canaria. The best 10 performing models out of 100 iterations of the SDM algorithm are presented (a–j) along with its average (k). Colour gradients represent the probability of occurrence for the species, from 0 (light blue) to 1 (dark yellow). Probability surfaces are presented on top of a topographical elevation model of Gran Canaria Island.

Generally, all present distribution models showed a similar distribution for *C. junoniae* in the island of Gran Canaria; this was expected given the ecology of the species. All models showed a higher probability of occurrence of the species in the north and northwest of Gran Canaria (Figure 3a–j). Towards the northeast, all models define an abrupt limit along the Tenoya ravine that runs along the island from the coast to the summit (Figure 3k). This ravine also marks changes in the landscape composition of the north of the island, from natural and rural areas with scattered settlements on the west side to a more agricultural and urbanised landscape to the east. The predicted potential distribution of the species is located on the north face of Gran Canaria (Figure 3k), at elevations that correspond to the potential distribution of pine forest transition with laurel and thermophilous forests. Many ravines expand the potential distribution of the species towards low‐elevation areas. These areas have the potential to act as corridors and microhabitats for the species (Figure 3k). This seems to be more relevant in the northeast side of the island where a large mosaic of potential habitat patches can be found (Figure 3k).

**FIGURE 4:**
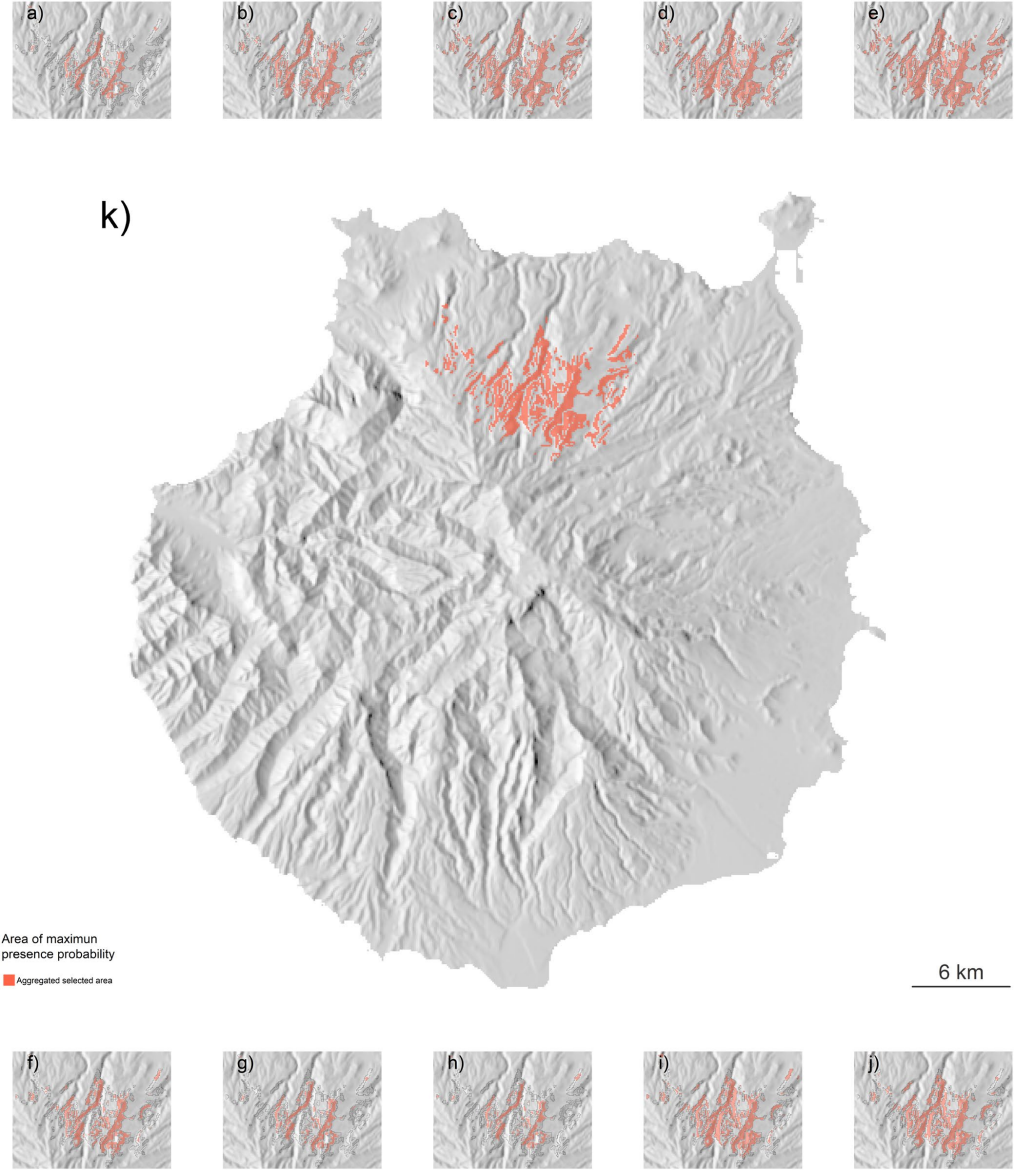
Prediction of the area of maximum probability of occupancy (AMPO) (red polygons) for the models regarding the historical distribution of *C. junoniae* (a–j). Details of the individual predictions for the 10 best models are presented in (panels a–j) along with the spatial combination of all Areas of Maximum Probability of Occurrence (AMPO) (k). The global AMPO (panel k) is the result of the combination of the individual models' AMPO spatial polygons. AMPO's are represented on top of a topographical elevation model of Gran Canaria Island.

**FIGURE 5:**
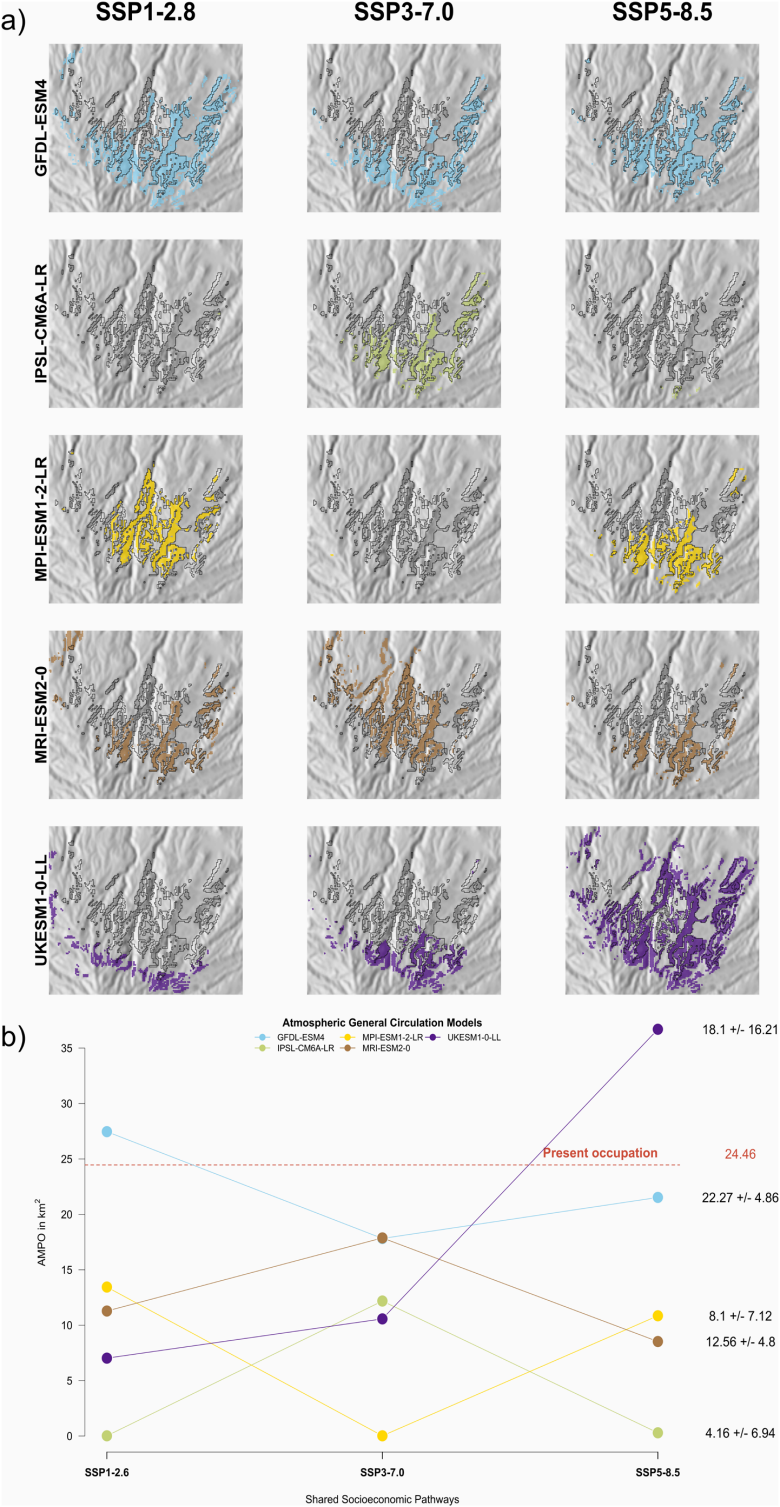
Predicted future Area of Maximum Probability of Occupancy (AMPO) for *C. junoniae* on Gran Canaria under different climate scenarios. (a) Spatial distribution of future AMPO: Coloured polygons represent AMPO under three Shared Socioeconomic Pathways (SSPs, columns) and five atmospheric circulation models (rows). The current AMPO (dark grey area, from Figure 4k) is shown for comparison. Future AMPO areas are calculated as the combined predictions from the 10 best‐performing models. (b) Changes in AMPO size: The *y*‐axis shows the absolute change in AMPO under each SSP (*x*‐axis) for different atmospheric models (coloured lines and points). The average change in AMPO (mean ± standard deviation) is shown on the right, compared to the current AMPO (red dashed horizontal line).

The current AMPO estimation was 24.46 km^2^, calculated over a flat area (Figures 4 and 5). From the Life+ Rabiche records, 49% fall within the limits of the current AMPO; similarly, 37% of records are in areas described by the models as of high probability of occurrence (probability higher than 0.65) and 81% are above the 0.5 probability threshold.

### 3.3 Future Scenarios

The 15 different future scenarios (three socioeconomic scenarios and five different atmospheric models) showed similar *C. junoniae* potential distribution patterns (Figure 5 and Supporting Information—S3). Four out of five climate change scenarios showed a decrease in the potential distribution of the species across the different socioeconomic conditions (Figure 5). In most cases, the predictions are concentrated in the north face of the island of Gran Canaria, with the Tenoya ravine still limiting the distribution of the species to the north‐west and small patches of the potential habitat persisting in the east‐north side of the island (Figure 5a).

On average, the AMPO for the species for the different shared socioeconomic pathways is predicted to halve compared to the predicted present AMPO (−51% for SSP1 2.6; −52% SSP3 7.0 and −36% for SSP5 8.5 respectively) across all climatic scenarios (Figure 5b). Similarly, there was a steep difference in the areas estimated from the different atmospheric models (Figure 5b). On average, shared socioeconomic pathways that used the IPSL atmospheric models showed a reduction of the area of occupancy of 82% (Figure 5b) whereas predictions based on the GFDL atmospheric model only showed a reduction in the area of occupancy of almost 9% (Figure 5b). The rest of the atmospheric scenarios showed a reduction in the area of occupancy between 26% and 66% when compared to the predicted present AMPO. In all cases but the combinations of SSP5‐8.2—UKESM1 and SSP1‐28—GFDL, the predicted areas of occupancy for the species were lower than the ones predicted for the present environmental conditions (Figure 5b). Generally, this reduction in area is caused by a loss of potential habitat in the median to low elevation as well as the northeast‐most areas of the original potential distribution (Figure 5a). In general, we predicted a slight change in the potential distribution of *C. junoniae* in elevation, moving towards the high‐elevation areas of the island (Figure 5a).

## 4 Discussion

The combination of Gbif as well as data derived from the reintroduction program of *C. junoniae* shows that the species has colonised most of its predicted potential distribution area. This habitat presents a high overlap with the potential distribution of plant formations such as laurel and thermophilous forests. However, predicted future climatic changes are likely to reduce the potential distribution area for the species on the island of Gran Canaria.

The results from the present potential distribution of *C. junoniae* on the island of Gran Canaria show that the new populations of the species have colonised large portions of its predicted potential habitat. Our results also show that the potential habitat of *C. junoniae* is strongly related to the different seasonality regimes of precipitation and, in a minor way, to the topographic and vegetation configuration of the landscape. Furthermore, the potential distribution of the species in Gran Canaria is located between the laurel and thermophilus forest potential distribution areas (del Arco Aguilar et al. 2010), which have been defined as preferred habitats for the species (Martín et al. 2020). However, the conservation status in Gran Canaria Island is poor, and only patches of these habitats remain (del Arco Aguilar et al. 2010). During the Life+ Rabiche, more than 220 ha of native forest have been planted to restore part of Gran Canaria native forests (Martín et al. 2020). Our results further remark on the importance of this forest restoration for *C. junoniae* . Furthermore, the impact of recovering and preserving these plant communities can have far‐reaching effects, providing fundamental ecosystem services and resiliency against climate change and biodiversity loss (Benayas et al. 2009; Newbold et al. 2019). Therefore, independently of *C. junoniae* , we recommend these restoration efforts to continue.

Furthermore, results suggest that future climate changes are likely to negatively affect *C. junoniae* in Gran Canaria Island by forcing changes in its distribution and reducing the amount of potential habitat for the species (e.g., Sekercioglu et al. 2008). However, the detected changes in area are solely attributed to predicted climate changes and do not consider other threats such as invasive alien species or land‐use changes (WWF 2020). These threats can have a great impact on the future distribution of the species but were not included in the model due to a lack of spatial information. In the case of Gran Canaria Island, feral cats ( *Felis silvestris* ) and black rats ( *Rattus rattus* ) pose a threat to biodiversity and therefore to the populations of *C. junoniae* (Hernández et al. 1999; Martín et al. 2000; Medina and Nogales 2009). Cats are widely distributed across the Canarian archipelago and their impacts on native species, including *C. junoniae* , are well documented (Martín et al. 2000; Medina and Nogales 2009). Other introduced alien predators, such as ferrets ( *Mustela putorius furo* ) or the California King snake ( *Lampropeltis getula californiae* ), both present in Gran Canaria, can also prey upon *C. junoniae* individuals or eggs. *C. junoniae* builds its nest in small cavities on the floor or walls covered by vegetation (Martín et al. 2000) and lays a single egg for reproductive events, making it especially sensitive to these alien invasive species. Including these potential threats in future models can greatly improve the predictive power and usefulness of SDMs for conservation and management.

This study, as any other species distribution model research, presents a series of limitations (Guisan and Zimmermann 2000; Pearson and Dawson 2003). Firstly, the amount and type of distribution data are limited. In our case, we have used a presence‐only approach, combining presence records of *C. junoniae* from various, imperfect datasets (Beck et al. 2014). Therefore, we must assume that such compilation of records is incomplete and that without a precise account of records and absences of the species, the results of our presence‐only approach are, at best, just approximations of the real habitat of the species (Pearce 2012). Second, the resolution and amount of environmental data limit the power of the analysis (Patiño et al. 2023). Islands are usually poorly covered by climatic and land‐cover/use remote data. Therefore, high‐resolution data is scarce for these areas. In our case, we were able to use high‐resolution topographic and climatic data, but the land‐use/cover data had to be downscaled to match the resolution of the rest of the variables. This makes the data compatible with the higher resolution one but limits its statistical inference. On top of that, we had to maintain land‐cover constant across the future climatic scenarios. Land‐use change is the main cause of biodiversity loss (WWF 2020) and the interactions between land use and climate change are likely to create synergies causing further biodiversity loss (Newbold 2018; Newbold et al. 2019). Additionally, invasive alien species are one of the main causes of biodiversity loss on islands (Spatz et al. 2022), but their distribution within these habitats is still poorly mapped. In this sense, our models are limited and probably overestimate the proportion of potential habitat remaining for the species under future scenarios of change. It is for these reasons that major efforts in mapping and forecasting these threats are needed to produce more reliable scenarios of biodiversity change.

## 5 Conclusion

Despite its limitations, from a conservation perspective, our results highlight the importance of developing long‐term monitoring and restoration strategies to ensure the success of the introduction process. The reintroduction of *C. junoniae* in Gran Canaria has the potential to favor the populations of other species through the restoration of its habitat (Branton and Richardson 2010) and, at the same time, restoring key ecosystem services and increasing the resiliency of the habitat against future climate changes (e.g., Mawdsley et al. 2009). In this regard, special attention must be put on the control of land‐use change and the expansion of alien invasive species. Additionally, our study emphasizes the need to expand the monitoring program of the species to locate and preserve microhabitats that might help the species to establish stable populations even in the face of climate change (e.g., Morelli et al. 2017).

This study presents the basis for the study, design, and long‐term planning of the conservation of *C. junoniae* on Gran Canaria Island. The results and methods used throughout this study remark on the value monitoring data have for the evaluation of conservation projects and, more particularly, for the evaluation of reintroduction programs. Although the monitoring data show that *C. junoniae* has been able to colonise most of its potential habitat, it is necessary to expand the monitoring to include potential clusters of microhabitats that have not been surveyed yet. Additionally, climate change is going to impose new challenges for the species in the form of range shifts and habitat reductions. To ensure the survival of *C. junoniae* in the future, it is necessary to undergo a long‐term habitat restoration and climate change attenuation strategy.

## Author Contributions

**Gonzalo Albaladejo‐Robles:** conceptualization (lead), data curation (lead), formal analysis (lead), investigation (lead), methodology (lead), software (lead), validation (lead), visualization (lead), writing – original draft (lead), writing – review and editing (lead). **José Manuel Caballero Fernández:** conceptualization (equal), data curation (equal), investigation (equal), writing – original draft (equal), writing – review and editing (equal). **Yarci Acosta Santana:** data curation (supporting), investigation (supporting), validation (equal), writing – review and editing (supporting).

## Conflicts of Interest

The authors declare no conflicts of interest.

## Supporting information


Data S1.

Data S2.

## Data Availability

Environmental information and GBIF presence records for the analysis are available in the links and references provided in the main text and Supporting Information—S1. The presence data and variable information used for the analysis are presented in the Supporting Information—S1. All analyses were carried out in R, and the base code is available as a GitHub repository at https://github.com/gonzaloalbaladejo/ee.columbajunoniae.
