# Supplementary material for: Present and Future of the White‐Tailed Laurel Pigeon (Columba junoniae) on Gran Canaria Island
Source: Ecol Evol. 2025 Jun 21;15(6):e71580. doi: 10.1002/ece3.71580 (PMC12181689; doi:10.1002/ece3.71580)
Supplement: Supplementary file 2 — Data S2. [file ECE3-15-e71580-s001.docx]

**Supplementary materials: Present and future of the White-Tailed Laurel Pigeon (*Columba junoniae*) in the island of Gran Canaria**

Table of Contents

S.1 Species records and background data 2

**Gbif records filtering and correction** **2**

**Monitoring records filtering** **2**

S.2 Environmental variables pre-processing

S.2.a Land-cover **3**

S.2.b Topographic information **4**

S.3 Background.data 5

S.4 Model-adjustments and selection 5

**S. 5 MaxEnt predictions for the combination of Shared Socioeconomic Pathways and atmospheric circulation models** .………………………………………………………………………………………….………………………………………..**13**

Tables and figures

Figures 11

Tables 8

# **S.1 Species records and background data**

## **Monitoring records filtering**

Despite the large number of observations (more than 9,000 georeferenced records) (Figure 1. C main text and Table S2), most of them were clustered around a reduced number of localities or observation stations (46% of the presence records are spread across 58 observation points) or individuals (3,000 observations for 34 individuals marked with radio or GPS transmitters) (Figure 1. C - Table S2). This asymmetry in the distribution of monitoring efforts can cause bias in the adjustment and later interpretation of the SDM (Syfert et al., 2013). To avoid as much as possible the effects of sampling bias from the monitoring data we; (1) Removed a total of 5,798 spatially duplicated records. Those belonging to the same observation points can cause inflation of the values of the environmental variables recorded at those locations (Guisan et al., 2017; Syfert et al., 2013); (2) Once stabilised, individuals tend to move across the landscape predictably, visiting more frequently their territories and feeding areas, causing spatial aggregation (e.g Veech 2010). This is particularly visible in the telemetry data from the Life+ Rabiche since the number of marked individuals is low and not representative of the whole population. Despite this, telemetry data accounts for almost 40% of the data. To reduce the effects a few individuals had on the total volume of distribution records, we performed an individual base spatially driven observation thinning at 200m, the equivalent of 2 grid cells on the environmental data (100m grid resolution). This way, we removed 3,009 records from the GPS data and 512 records from the radio-tracking data. In total, we removed 9,321 (96%) of the Life+ Rabiche records (Table S2).

## **Gbif records filtering and correction**

The precision and reliability of the information varies greatly within the platform (Beck et al., 2014). Therefore, a strict filtering of the observations is needed. As with the monitoring data, we follow a hierarchical filtering approach. First, we removed all the records that presented spatial or metadata issues. These issues are marked on the “issues” field provided along each GBIF record (Table S1 - Table S2). Second, we removed all the points with erroneous information, conflicted metadata, and erroneous spatial distribution using the R-package *coordinate cleaner* version 2.0-20 (Zizka et al., 2019). Third, since some GBIF data is not professionally collected, we used the regional atlas of the species to remove the observations outside the areas in which the species has been detected by conventional normalized transects or observation points. This atlas is composed by the Spanish Ornithological Society (SEO-Birdlife, <https://seo.org>) and despite not providing the precise location of the individuals detected, it gave us information about the approximate distribution of the species across a UTM grid of 10 km^2^ that covers the entire archipelago (Romero-Rodriguez, 2022). GBIF records were retrieved using the R package *rgbif* version 3.7.7 (Chamberlain et al., 2023).

As a result of the filtering of both the records from the Life+ Rabiche monitoring program and the presence data from GBIF, we retain a total of 633 records (Table S2). Further testing into the structure of the presence data revealed very weak (below 0.15) but significant spatial autocorrelations between the environmental variables (Figure S1).

# **S.2 Environmental variables pre-processing**

## **S.2.a Land-cover**

The land use information from the European Space Agency Climate Change Initiative Land Cover (ESA CCI LCP, <http://www.esa-landcover-cci.org>) version 2.0.7 (ESA-LC hereafter) contains 36 different land use categories and covers the whole planet at a spatial grid resolution of 300 m. Categorical variables tend to cause overfitting problems on SDMs and are usually not that informative, compared to continuous variables. For this study, we have transformed the categorical land-use data into 14 continuous land-cover variables that describe the overall plant functional types associated with each land-use cover (Li et al., 2017; Harper, 2023). Transformation was carried out using a cross-walking table between the discrete land-use metrics and plant functional types (Li et al., 2017); broadleaf evergreen trees; broadleaf deciduous trees; needleleaf evergreen trees; needleleaf deciduous trees; broadleaf evergreen shrubs; broadleaf deciduous shrubs; needleleaf evergreen shrubs; needleleaf deciduous shrubs; natural grass; bare-soil; cropland; and urban (Li et al., 2017). Other classes not included in the analysis included permanent bodies of water snow and ice (Li et al., 2017). The final plant functional types reflect the percentage of total cover, between 0-1, of that plant type for a given pixel. Similar to Li et al (2017) and Albaladejo et al., (2022), we grouped the plant functional types covers according to our interests. We grouped all the tree cover data under a unique land-cover variable (tree), and all the shrubs cover types into a single layer (shrub). This resulted in a final land-cover dataset containing 6 variables: tree, shrub, cropland, grass, urban, and bare soil. We adapted the resolution of the land-cover information to 100 m using bilinear interpolation (e.g. Latombe et al., 2018).

## **S.2.b Topographic information**

Most topographic variables were calculated de novo from the reference 25 m resolution elevation data (<http://centrodedescargas.cnig.es/CentroDescargas/index.jsp>). Roughness was calculated as the mean absolute difference between our target pixel and the 8 surrounding adjacent pixels (Wilson et al., 2007). We calculate the slope using the moment of elevation vectorial gradient, the magnitude of such momentum is what determines the slope (Fleming & Hoffer, 1979; Ritter, 1987). In the case of the terrain aspect, this was calculated from the angle between the slope and the horizontal plane (Fleming & Hoffer, 1979; Ritter, 1987). Flow direction was calculated as the difference in elevation between adjacent cells (Wilson et al., 2007). The elevation was extracted directly from the DEM at a grid resolution of 25 m. All variables were upscaled to 100 m resolution using bilinear interpolation (e.g. Chakraborty et al., 2021).

# **S.3 Background data**

Although MaxEnt is considered a presence-only SDM method, the algorithm still needs to generate background information to compare the presence data against a sample of background points. By default, MaxEnt creates a set of 10,000 randomly distributed points across the study area. However, how these points are created and their distribution can impact the accuracy and performance of our final model (Barbet-Massin et al., 2012). This is particularly important when imperfect sampling occurs, or the presence records are highly aggregated (both cases present in our data). To evaluate the impact of the background data on our SDM models, as well as to select the best set of background points, we created these points using four different methods across the distribution area of *C. junoniae* (Figure 1.a); (1) background points were randomly distributed (Figure 1. a-b); (2) background points based on *C. junoniae* presence points, where a density matrix is built based on the distribution of the species and background points are distributed accordingly to this density matrix (Barbet-Massin et al., 2012) (Figure S2. b); (3) background points distributed according to sampling bias. For this, we gathered all the Gbif data for all the species in the Canarian archipelago and built a matrix of total observations to guide the distribution of background points (Figure S2. c); (4) using that same Gbif data, background points distributed according to a density kernel of observations (Figure S2. d). Each set of background points is formed out of 10.000 points, the default number of background points MaxEnt uses (Phillips et al., 2006).

# **S.4 Model adjustments and selection**

Even though MaxEnt can resolve problems with highly dimensional data, a variable selection is needed to remove any potential autocorrelations and redundancy from our models. Variable autocorrelation can add heteroscedasticity and overfitting to our model (Pradhan, 2016), rendering their predictions inaccurate. So, to reduce the number of variables and prevent problems of overfitting and autocorrelation we run a variance inflation factor analysis (VIF) on our environmental variables (Miles, 2014). FIV is a measure of variable collinearity, as a rule of thumb VIF values between pairs of variables below 5 represents low variable collinearity (O’Brien, 2007). Values above this threshold mark moderate to high variable collinearity. In the first selection step, we calculated the VIF for all the variables, the ones that presented values of VIF above 5 were dropped, this process was repeated until all remaining variables presented values of VIF below 5 (Table S3. a). In a second selection round, we calculated Spearman correlation coefficients (Quinn & Keough, 2002) of the remaining variables. In the cases where pairwise correlation was higher than 0.7, we selected the variables that we considered were more important for the explanation of the distribution of *C. junoniae* and excluded the others (e.g Patiño et al., 2023) (Table S3. b). With this filtering, we reduced our pool of environmental variables from 29 to 13: isothermality (bio_03), temperature seasonality (bio_04), mean temperature of the driest quarter (bio_09), precipitation of the driest month (bio_14) and precipitation seasonality (bio_15), precipitation of the warmest quarter (bio_18), percentage of grass and tree cover (Grass and Tree), percentage of cropland and urban cover (Crop and Urban), terrain aspect, flow direction (flowdir), and terrain slope.

After this initial selection of variables, we run a series of preliminary MaxEnt models to evaluate the effects of the variables and the type of background information on the probability of occurrence of *C. junoniae*. For this, we separate the background and presence data into train and test subsets (80% and 20% of the records respectively). To evaluate the contribution of the different environmental variables to the models we run a JackKnife test (Phillips et al., 2006) along with MaxEnt. The JackKnife test removes a variable at a time from the model, runs the model, and then adds the variable again. By doing this, we can evaluate variable importance and impact on model performance (Phillips et al., 2006). After this process, we selected the most relevant variables for the distribution of *C. junoniae* and the set of background data that returned the overall best fit for the SDM.

As a result of these preliminary models, we selected the set of background data generated at random (Figure S2. a) and 9 environmental variables: seasonal temperature and precipitation, precipitation of the driest month, percentage of herbaceous and tree cover, percentage of crop coverage, aspect, and slope (Figure 2. B main text). Tree cover and elevation were also added to the final pool of variables due to their potential importance in the distribution and habitat selection of the species. To prevent variable autocorrelation and collinearity, VIF and correlation tests were calculated again for the final set of variables. In this case, all VIF values were below 5 and Spearman-rank correlation values were below 0.7 (Table S2).

All analysis and data preprocessing was carried out in R-project version 4.3.1 (R-core team, 2023). The spatial information was corrected and projected to the UTM reference coordinate system REGCAN95 (EPGS: 4083) using the R package “*sf*” version 1.0-13 (Pebesma, 2018) and the R package “*terra*” version 1.7-37 (Hijmans, 2023). These packages were also used to perform other spatial processing such as background sampling. Gbif information was downloaded using the R package “*rgbif*” version 3.7.7 (Chamberlain et al., 2023) and filtered using the R package “*CoordinateCleaner*” version 2.0-20 (Zizka et al., 2019). Distance matrices for the calculation of density kernels were built using a combination of custom-made functions and the R package “*MASS*” version 7.3-60 (Venables & Ripley, 2002). Mantel spatial correlogram analysis was performed using a function adapted from the R package “*ecodist*” version 2.0.9 (Goslee & Urban 2007). Variable selection using the VIF and cross-correlations were implemented using the R packages “*car*” version 3.1-2 (Fox & Weisberg, 2019) and “*usdm*” version 1.1-18 (Naimi et al., 2014). All MaxEnt models were fitted and checked using the R package “*dismo*” version 1.3-14 (Hijmans et al., 2023).

# **Tables**

**Table S1**. Global Biodiversity Information Facility (Gbif) recorded issues used to filter the point information. Codes, issue names (Issue), and their descriptions are presented as in the Gbif database along with the number of times these issues are recorded in the original *C. junoniae* records (N records).

| **Code** | **Issue** | **Description** | **N records** |
| --- | --- | --- | --- |
| bri | BASIS_OF_RECORD_INVALID | The given basis of record is impossible to interpret or seriously different from the recommended vocabulary. | 5 |
| cdround | COORDINATE_ROUNDED | Original coordinate modified by rounding to 5 decimals. | 489 |
| incomis | INSTITUTION_COLLECTION_MISMATCH | The collection matched doesn't belong to the institution matched. | 1 |
| inmafu | INSTITUTION_MATCH_FUZZY | The given institution was fuzzily matched to a GrSciColl institution. | 1 |

**Table S2**. Relation of raw and filtered (Clean) *C. junoniae* records for each island within its distribution area. These records included both GBIF and Life+ Rabiche data.

|  | **Tenerife** | **La Gomera** | **EL Hierro** | **La Palma** | **Gran Canaria** | **Total records** |
| --- | --- | --- | --- | --- | --- | --- |
| Clean | 172 | 77 | 1 | 61 | 322 | **633** |
| Raw | 759 | 224 | 1 | 270 | 9,712 | **10,966** |

**Table S3**. Variance inflation factor (VIF) matrix of the set of environmental variables considered for the modelling of the potential distribution of *C. junoniae* in the Canary Islands. The VIF is iteratively calculated for all the environmental variables with the variables with VIF values below 5 being removed one at a time. Selected variables are marked in **bold**.

| **Variables** | **0** | **1** | **2** | **3** | **4** | **5** | **6** | **7** | **8** | **9** | **10** | **11** | **12** |
| --- | --- | --- | --- | --- | --- | --- | --- | --- | --- | --- | --- | --- | --- |
| **aspect** | 1 | 1.1 | 1.1 | 1.1 | 1.1 | 1.1 | 1.1 | 1.1 | 1.1 | 1.1 | 1.1 | 1.1 | 1.1 |
| Bare | 10 | 10.4 | 9.6 | 9.7 | 10.0 | 9.8 | 8.9 | 9.5 | 8.8 | 10.0 | 8.9 | 8.8 | 9.4 |
| bio_01 | 309 | 312.1 | 306.9 | 281.8 | 256.7 |  |  |  |  |  |  |  |  |
| bio_02 | 450 | 444.3 |  |  |  |  |  |  |  |  |  |  |  |
| **bio_03** | 29 | 30.0 | 12.1 | 11.4 | 11.6 | 11.3 | 10.6 | 9.4 | 4.8 | 4.9 | 4.8 | 4.3 | 1.8 |
| **bio_04** | 105 | 101.0 | 101.1 | 51.2 | 52.8 | 39.2 | 30.2 | 28.1 | 20.8 | 21.4 | 21.9 | 11.6 | 4.8 |
| bio_05 | 500> | 142.4 | 43.9 | 38.9 | 37.6 | 38.7 | 22.1 | 21.7 | 21.0 | 21.9 | 21.8 | 16.6 |  |
| bio_06 | 500> | 295.1 | 85.9 | 73.4 | 72.5 | 69.9 | 47.2 | 46.8 |  |  |  |  |  |
| bio_07 | 500> |  |  |  |  |  |  |  |  |  |  |  |  |
| bio_08 | 38 | 37.2 | 34.8 | 35.9 | 35.1 | 35.1 | 27.4 | 29.5 | 24.0 | 24.8 | 23.6 |  |  |
| bio_09 | 20 | 20.2 | 19.1 | 19.9 | 18.7 | 19.3 | 16.0 | 15.9 | 12.2 | 12.4 | 12.1 | 11.0 | 6.5 |
| bio_10 | 332 | 328.0 | 321.4 | 272.0 | 248.8 | 116.1 |  |  |  |  |  |  |  |
| bio_11 | 428 | 401.9 | 373.4 |  |  |  |  |  |  |  |  |  |  |
| bio_12 | 140 | 147.5 | 138.5 | 138.8 | 84.0 | 85.4 | 77.3 |  |  |  |  |  |  |
| bio_13 | 186 | 200.8 | 190.8 | 201.7 | 47.9 | 47.0 | 46.2 | 13.2 | 12.7 | 12.2 | 11.9 | 10.6 | 10.5 |
| **bio_14** | 32 | 32.1 | 30.5 | 30.2 | 30.7 | 31.1 | 29.2 | 29.7 | 27.8 | 29.8 | 3.7 | 3.6 | 3.5 |
| **bio_15** | 6 | 6.1 | 6.0 | 5.9 | 6.0 | 6.0 | 5.9 | 3.7 | 3.7 | 3.7 | 3.4 | 3.5 | 3.4 |
| bio_16 | 335 | 356.7 | 331.7 | 344.5 |  |  |  |  |  |  |  |  |  |
| bio_17 | 43 | 42.7 | 41.1 | 41.2 | 40.9 | 42.3 | 38.7 | 36.3 | 34.7 | 36.8 |  |  |  |
| bio_18 | 3 | 3.3 | 3.2 | 3.1 | 2.9 | 3.0 | 3.0 | 2.9 | 2.9 | 2.9 | 2.7 | 2.6 | 2.6 |
| bio_19 | 16 | 17.5 | 17.3 | 16.6 | 16.4 | 17.0 | 16.0 | 13.5 | 13.1 | 12.4 | 12.3 | 10.8 | 11.1 |
| **Crop** | 2 | 2.4 | 2.3 | 2.4 | 2.3 | 2.4 | 2.3 | 2.2 | 2.1 | 2.3 | 2.2 | 2.1 | 2.3 |
| Elevation | 25 | 24.3 | 21.9 | 21.3 | 19.9 | 19.1 | 14.6 | 15.2 | 14.1 | 14.3 | 13.9 | 12.3 | 11.2 |
| flowdir | 1 | 1.2 | 1.2 | 1.2 | 1.2 | 1.2 | 1.2 | 1.2 | 1.2 | 1.1 | 1.1 | 1.1 | 1.1 |
| **Grass** | 7 | 7.4 | 6.7 | 6.8 | 7.1 | 6.7 | 6.4 | 6.8 | 6.3 | 6.8 | 6.3 | 6.2 | 6.7 |
| Roughness | 36 | 37.5 | 36.7 | 37.4 | 37.3 | 36.6 | 37.3 | 35.8 | 37.1 |  |  |  |  |
| Shrub | 2 | 2.3 | 2.2 | 2.3 | 2.3 | 2.2 | 2.2 | 2.2 | 2.2 | 2.3 | 2.2 | 2.2 | 2.2 |
| **slope** | 19 | 20.4 | 19.7 | 20.2 | 19.7 | 20.3 | 20.3 | 18.0 | 19.0 | 14.2 | 14.2 | 13.2 | 13.2 |
| T.rough_index | 25 | 28.8 | 27.8 | 30.4 | 28.5 | 26.7 | 28.2 | 28.4 | 28.6 | 14.4 | 14.3 | 13.3 | 13.5 |
| **Tree** | 4 | 4.3 | 4.1 | 4.2 | 4.2 | 4.1 | 4.0 | 4.0 | 3.8 | 3.9 | 3.9 | 3.8 | 3.8 |
| Urban | 2 | 1.8 | 1.8 | 1.7 | 1.8 | 1.7 | 1.7 | 1.7 | 1.7 | 1.8 | 1.7 | 1.7 | 1.7 |

**Table S4.** Correlation matrix for the environmental variables considered (first selection) for the modelling of the potential distribution of *C. junoniae* on the Canarian archipelago.

|  | bio_03 | bio_04 | bio_09 | bio_13 | bio_14 | bio_15 | bio_18 | Crop | Grass | Shrub | Tree | Urban | slope | aspect |
| --- | --- | --- | --- | --- | --- | --- | --- | --- | --- | --- | --- | --- | --- | --- |
| bio_04 | 0.26 | -- | -- | -- | -- | -- | -- | -- | -- | -- | -- | -- | -- | -- |
| bio_09 | 0.13 | 0.04 | -- | -- | -- | -- | -- | -- | -- | -- | -- | -- | -- | -- |
| bio_13 | 0.29 | 0.41 | -0.42 | -- | -- | -- | -- | -- | -- | -- | -- | -- | -- | -- |
| bio_14 | 0.28 | -0.01 | -0.08 | 0.47 | -- | -- | -- | -- | -- | -- | -- | -- | -- | -- |
| bio_15 | -0.41 | 0.01 | -0.01 | -0.31 | -0.74 | -- | -- | -- | -- | -- | -- | -- | -- | -- |
| bio_18 | 0.05 | -0.24 | -0.27 | 0.29 | 0.68 | -0.56 | -- | -- | -- | -- | -- | -- | -- | -- |
| Crop | 0.16 | -0.08 | 0.11 | 0.03 | 0.25 | -0.27 | 0.2 | -- | -- | -- | -- | -- | -- | -- |
| Grass | 0.28 | 0.52 | 0.1 | 0.21 | -0.02 | 0.07 | -0.09 | -0.14 | -- | -- | -- | -- | -- | -- |
| Shrub | 0.33 | 0.17 | -0.1 | 0.44 | 0.51 | -0.51 | 0.31 | 0.12 | 0.01 | -- | -- | -- | -- | -- |
| Tree | 0.24 | 0.24 | -0.27 | 0.65 | 0.43 | -0.4 | 0.23 | -0.08 | -0.06 | 0.55 | -- | -- | -- | -- |
| Urban | 0.1 | -0.13 | 0.11 | -0.11 | -0.03 | -0.04 | 0.04 | -0.03 | -0.15 | -0.11 | -0.1 | -- | -- | -- |
| slope | 0.19 | 0.24 | -0.09 | 0.4 | 0.24 | -0.2 | 0.16 | -0.06 | 0.38 | 0.21 | 0.29 | -0.13 | -- | -- |
| aspect | 0.04 | 0 | -0.07 | 0.02 | 0.01 | -0.02 | 0.02 | -0.08 | 0.04 | -0.01 | 0.05 | 0 | 0.05 | -- |
| flowdir | 0.06 | -0.1 | -0.05 | 0.08 | 0.18 | -0.2 | 0.17 | 0.1 | -0.1 | 0.09 | 0.09 | -0.01 | 0.01 | -0.2 |

# **Figures**

**
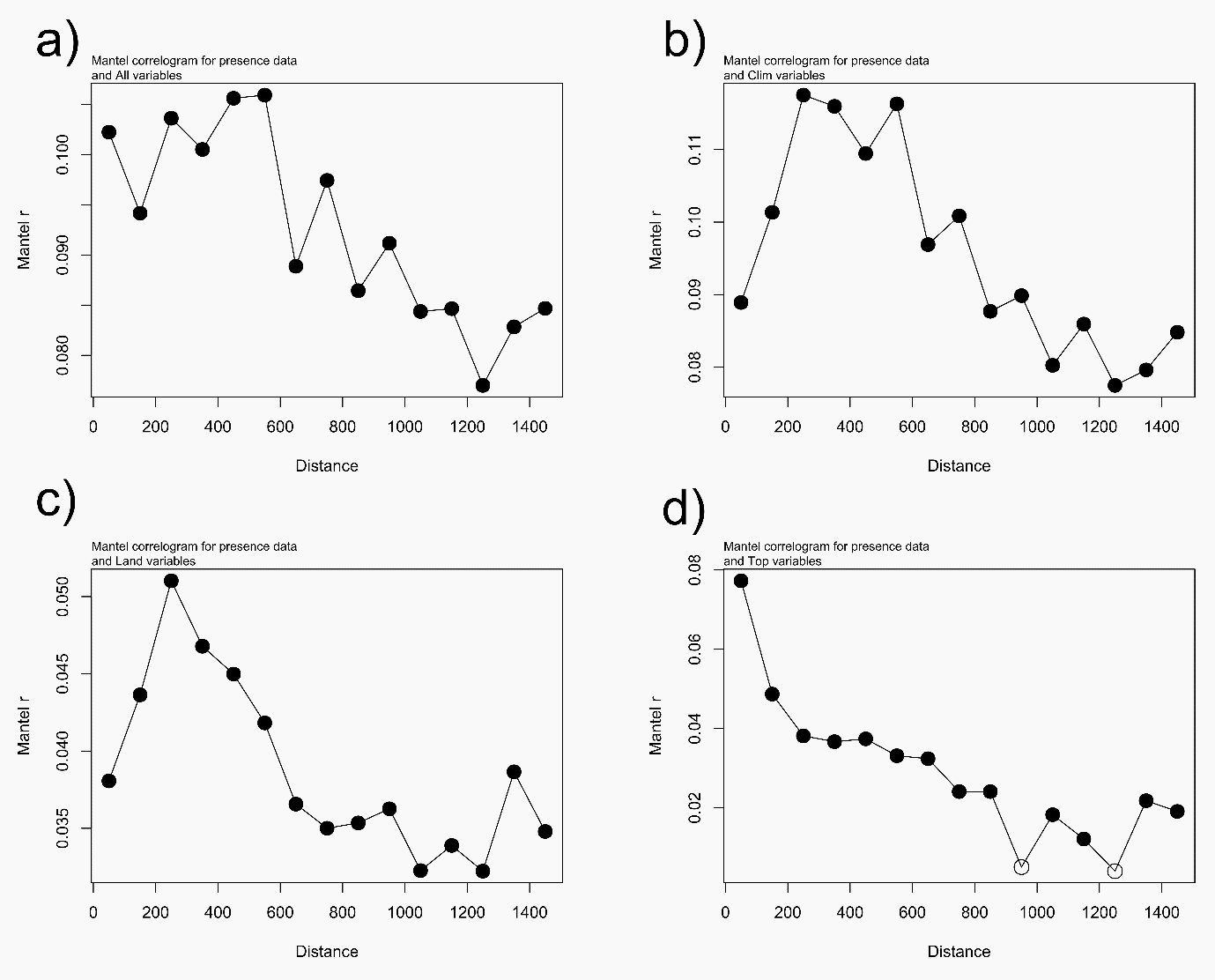
**

**Figure S1**. Mantel correlogram of the presence records considering the variation within all the environmental variables (a); only the climatic variables (b); only the land cover variables (c); and the topographical variables (d). Filled circles represent statistically significant values of spatial autocorrelation (p-value < 0.05) whereas empty circles represent non-significant correlation values.


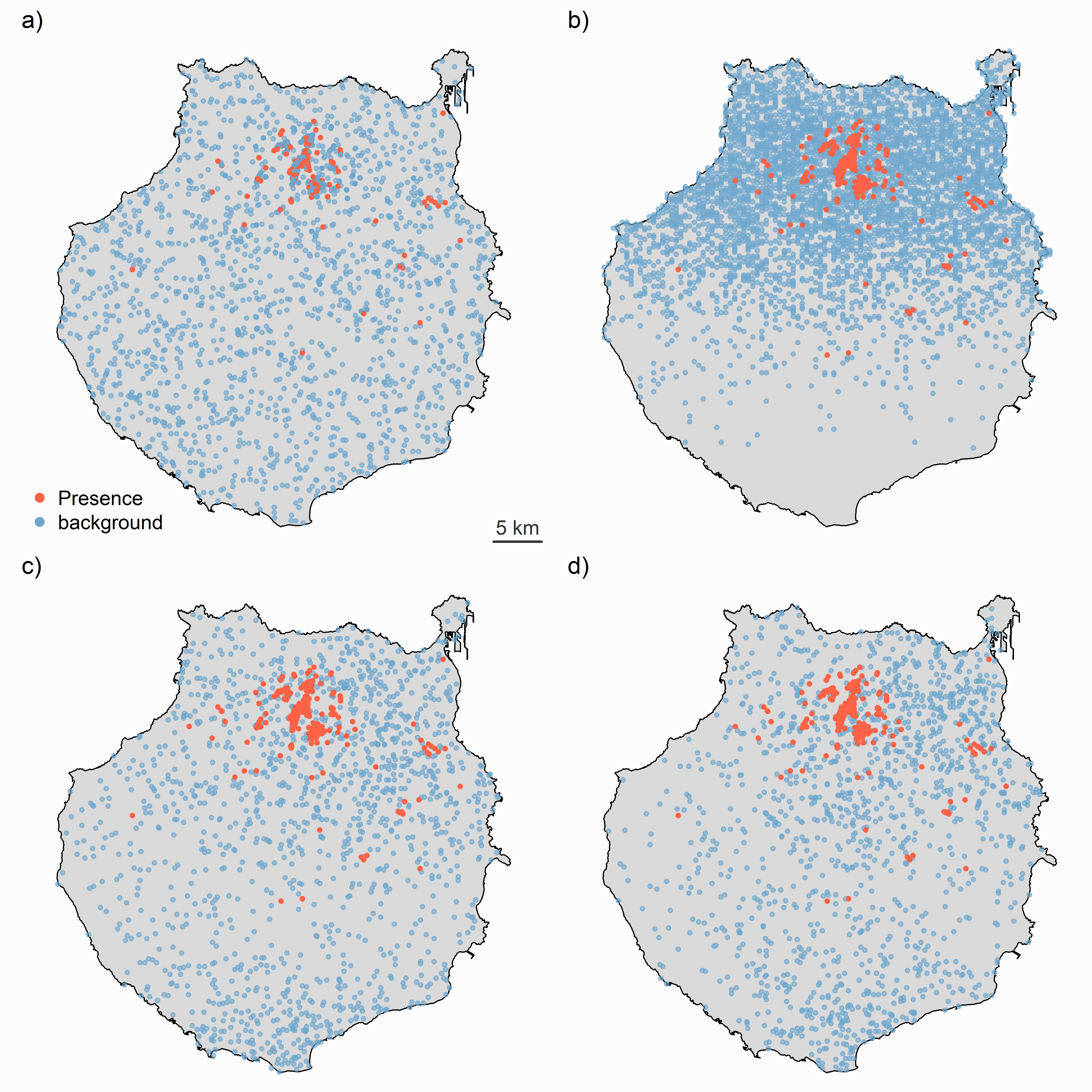


**Figure S2**. Distribution points of *C. junoniae* in the island of Gran Canaria (red dots) along with background points (blue dots) created using different approaches; **a**, randomly generated background points; **b**, background points generated using a density kernel of presence data; **c**, background points generated following a density kernel created using all the available Gbif records (all species); and **d**, distribution of background data based on the raw number of Gbif records. A total of 10,000 background data were generated across the whole range of the species (see Figure 1. A in the main text).

# **S. 5 MaxEnt predictions for the combination of Shared Socioeconomic Pathways and atmospheric circulation models**

This section contains the potential distribution of *C. junoniae* for the 15 different scenarios of future climate change. The figures presented in this section are composed of the 10 best-performing MaxEnt models and their predictions over Gran Canaria Island (panels a to j) along with the average of these values (panel k). The combination of SSP and atmospheric models is presented in the bottom left corner of panel K in each figure.

**
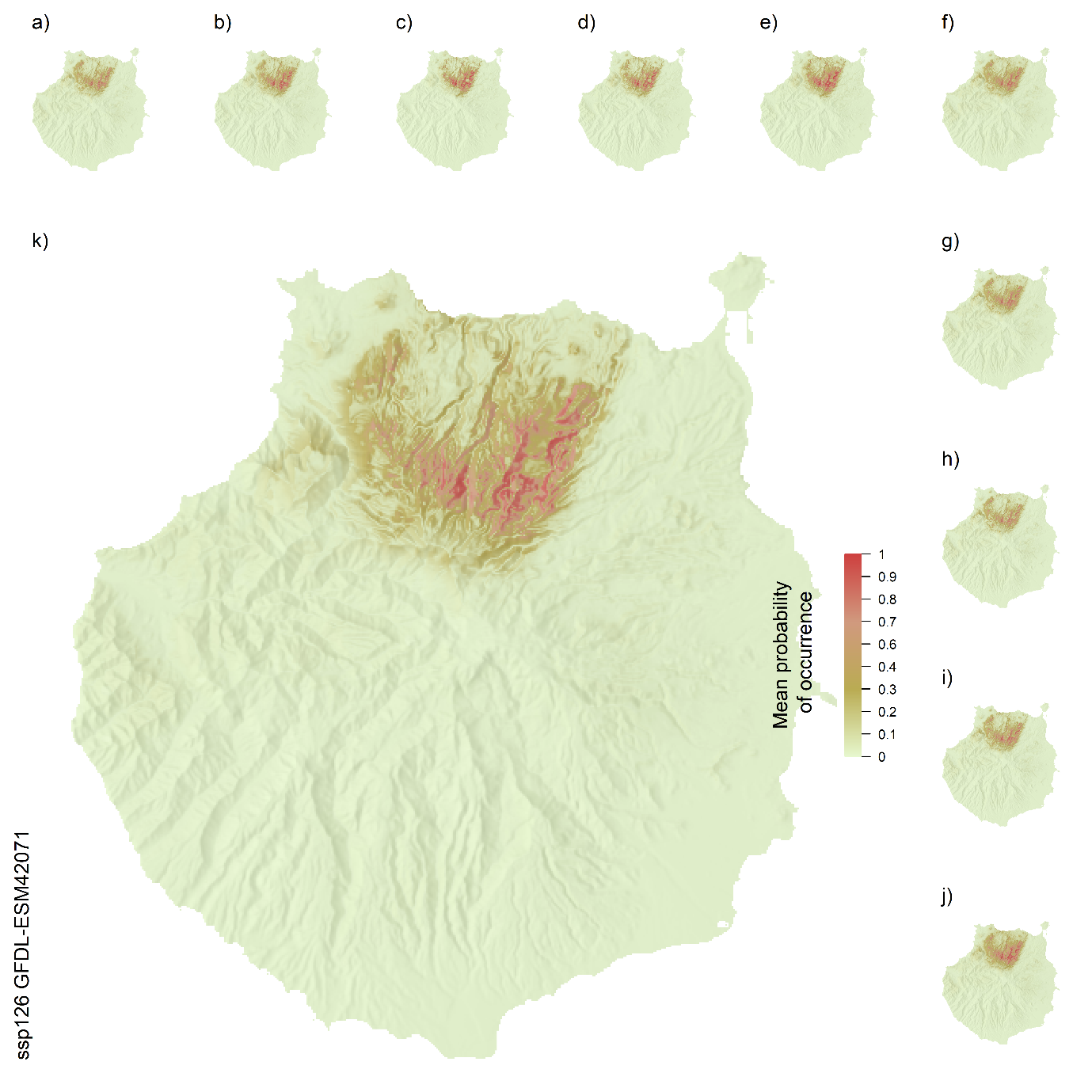
**

**
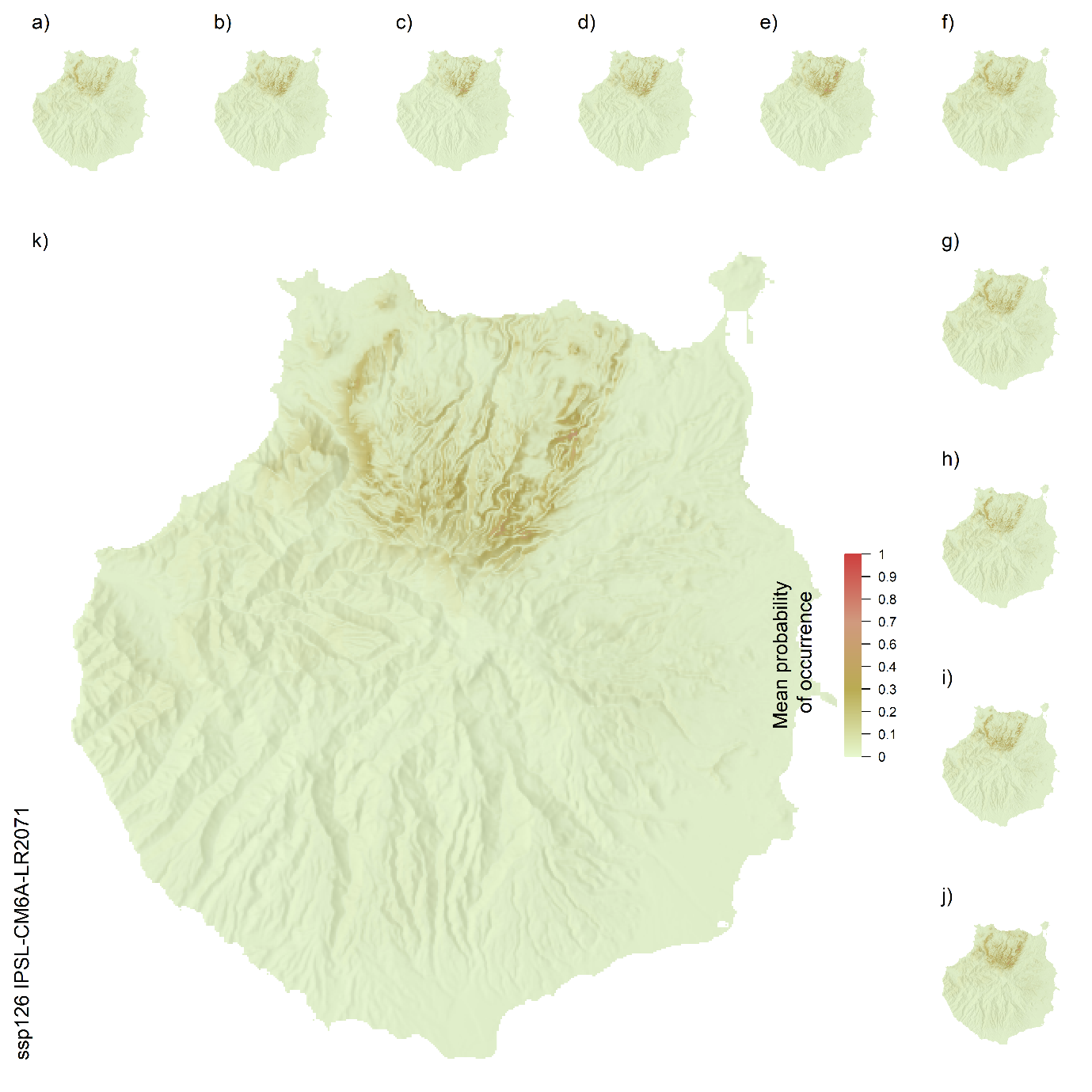

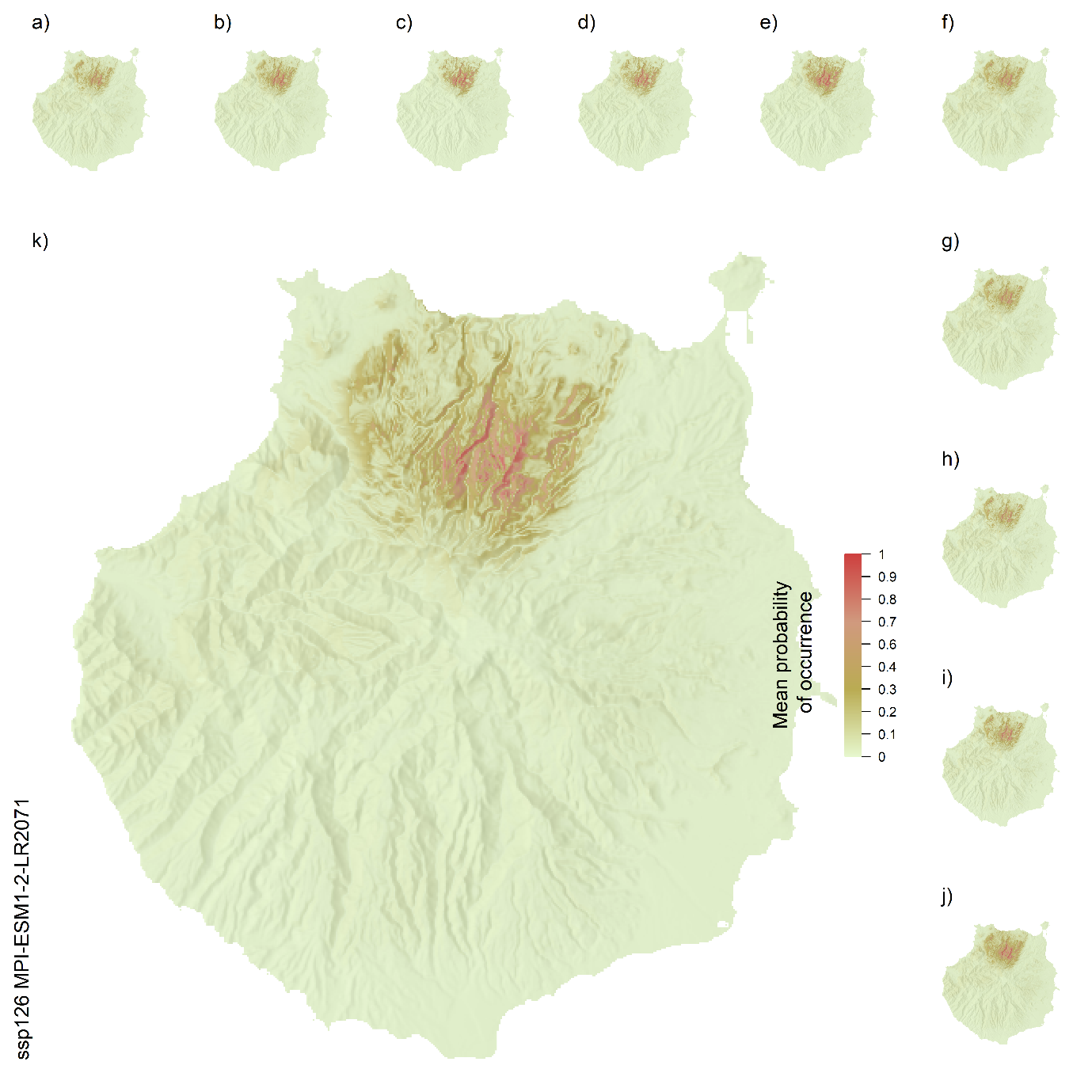

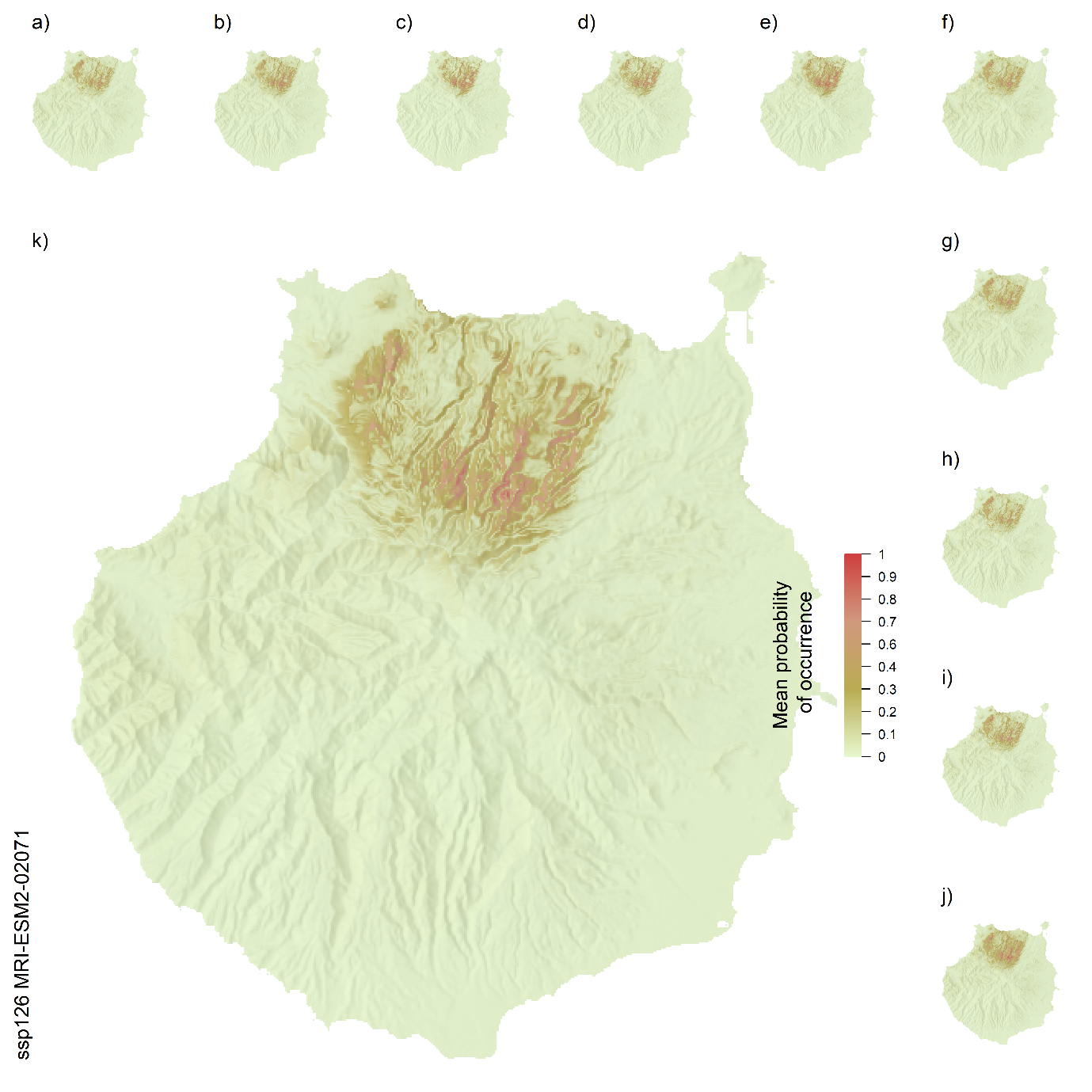

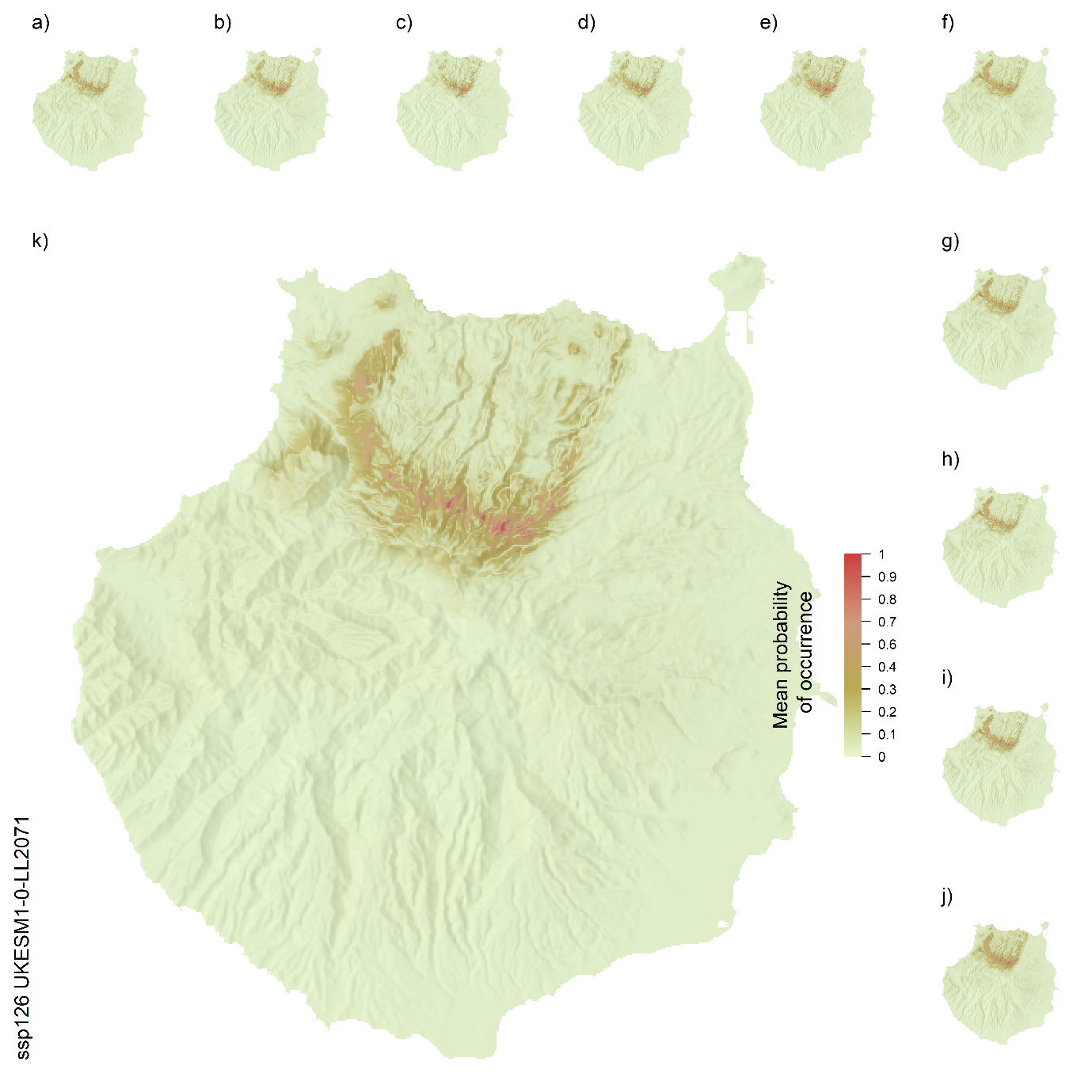
**

**
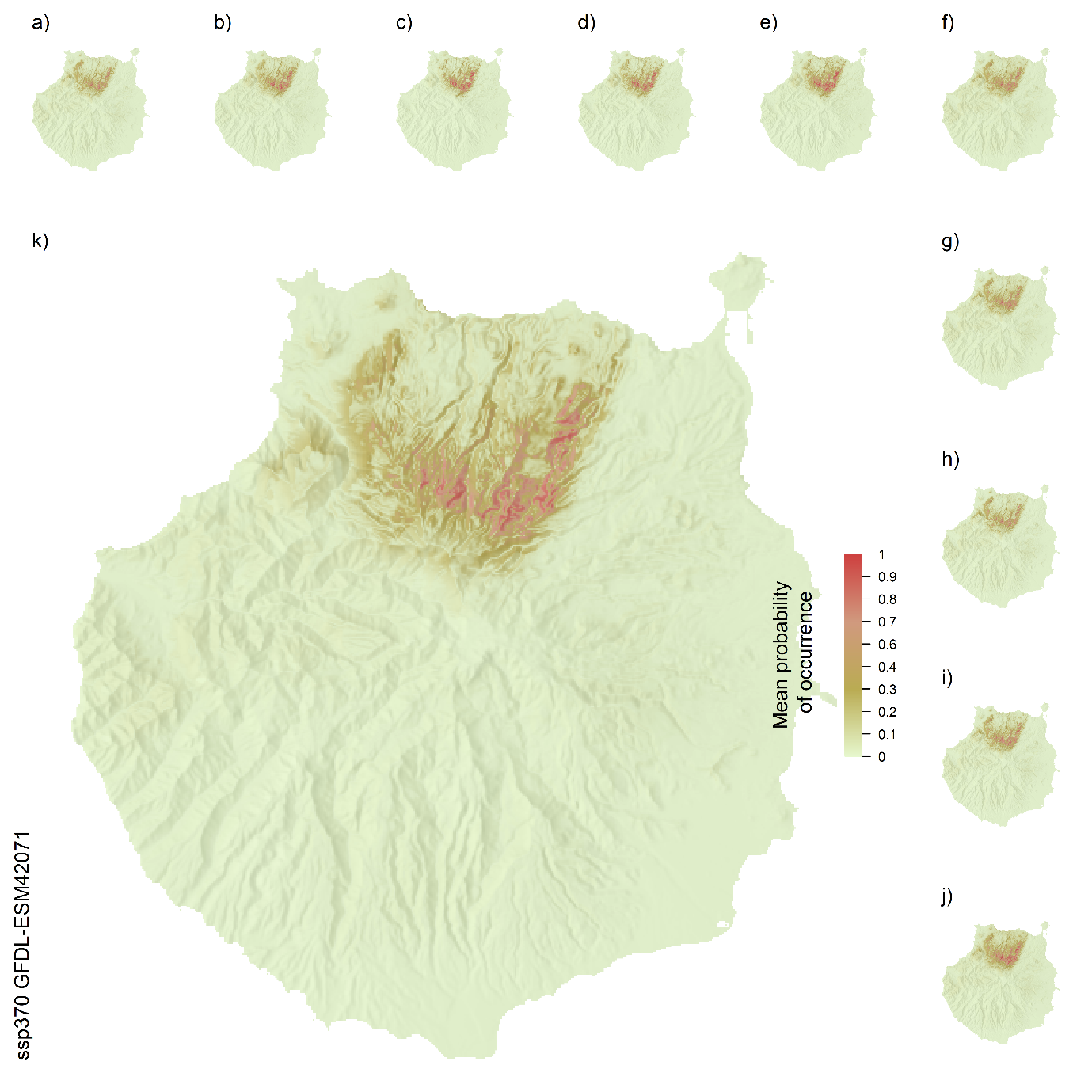

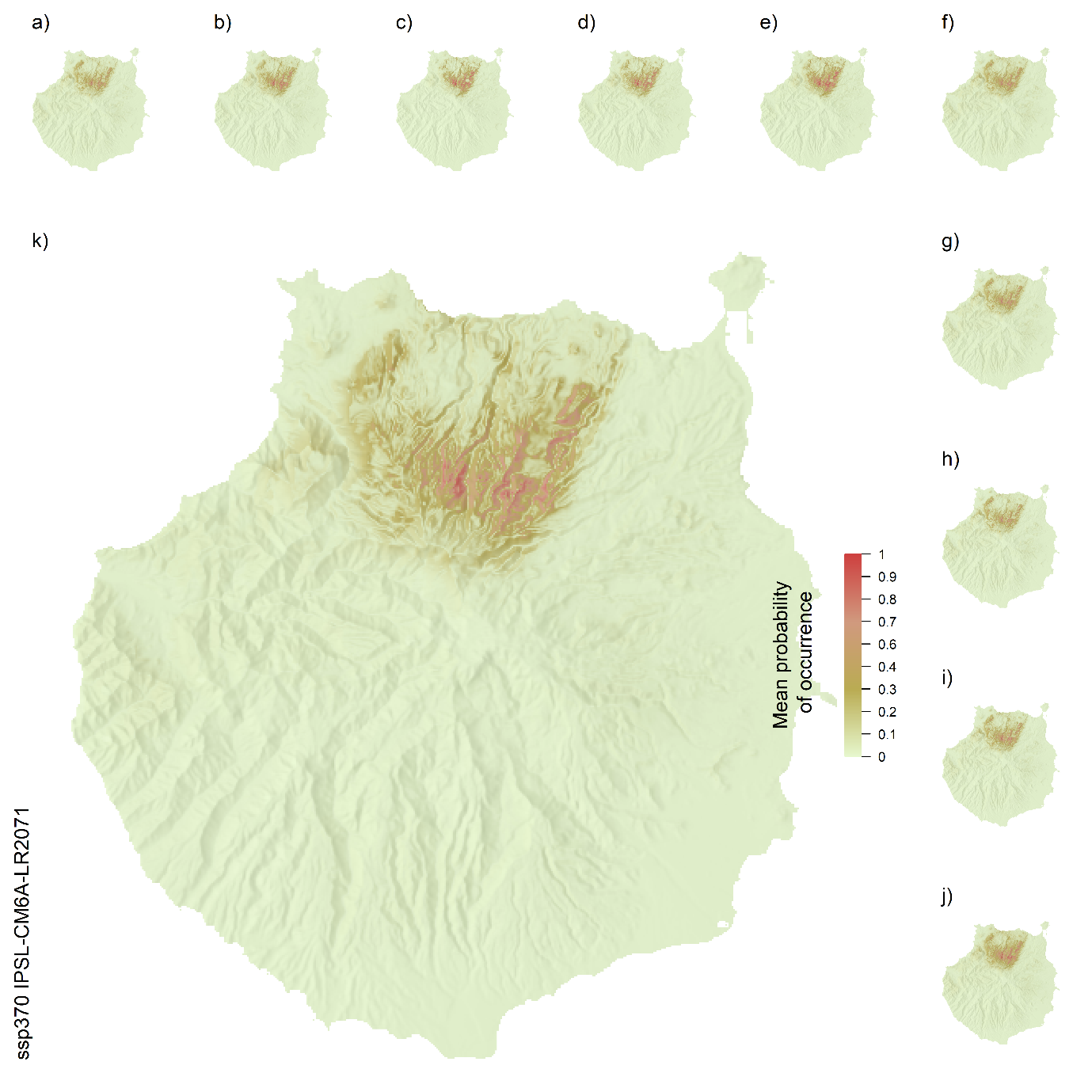

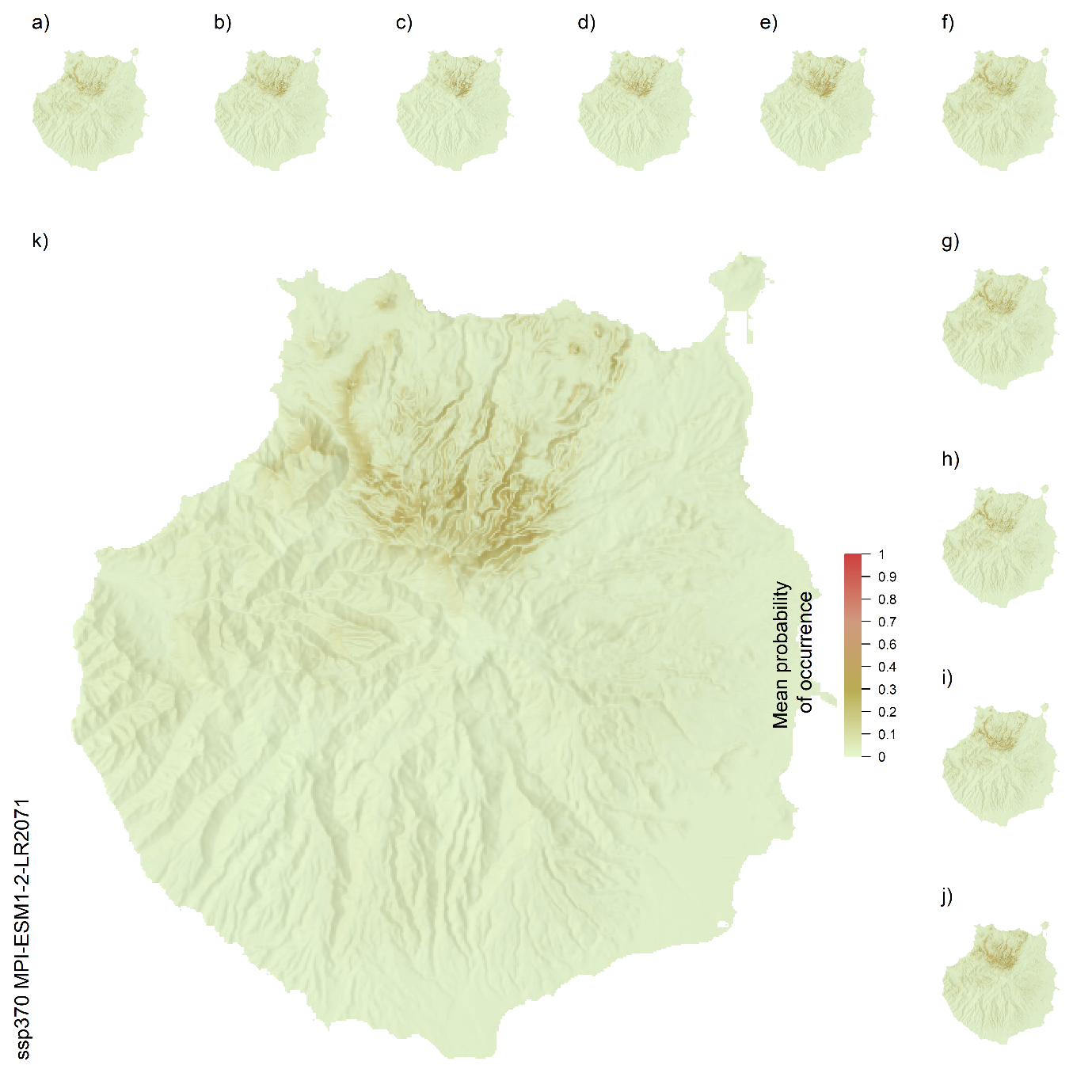

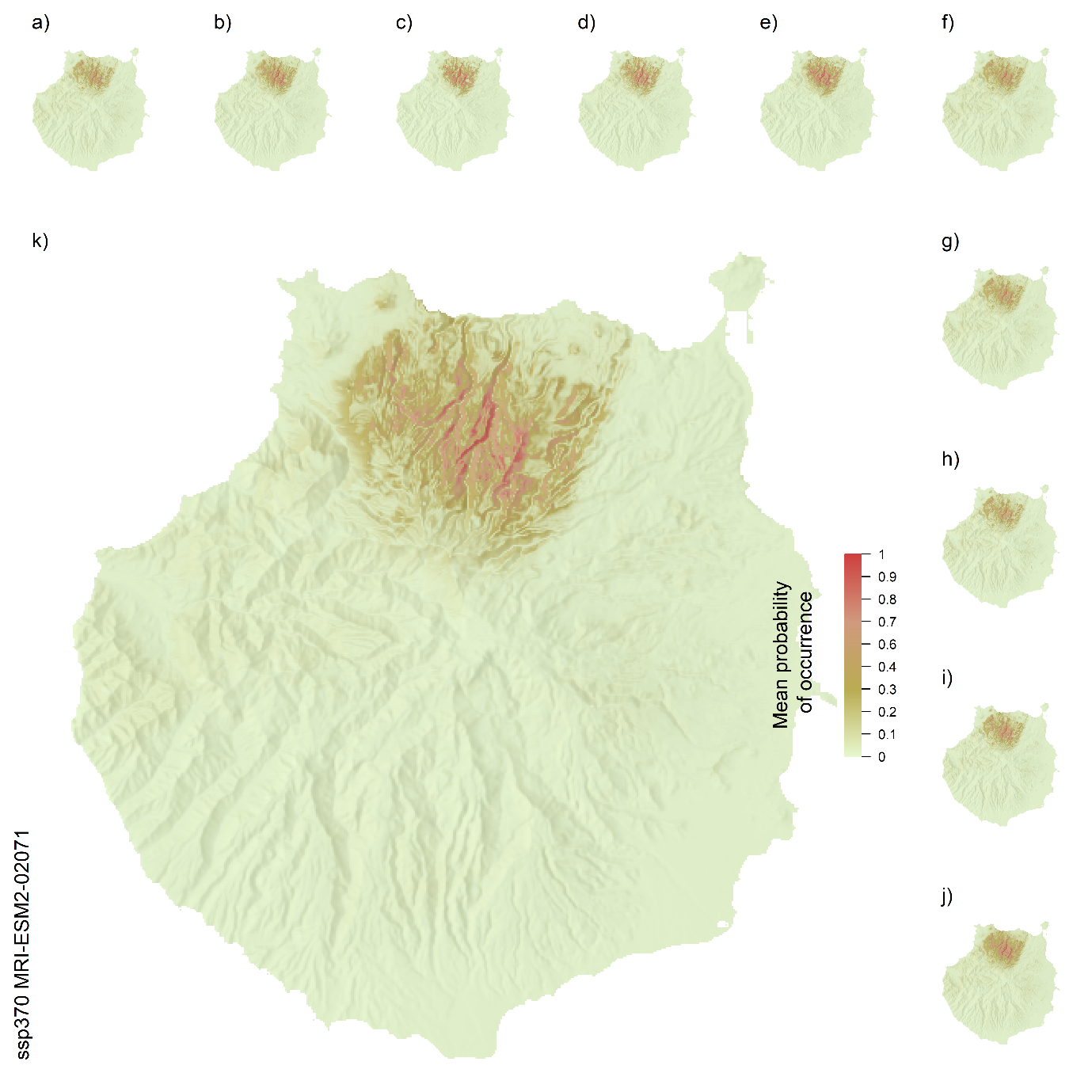

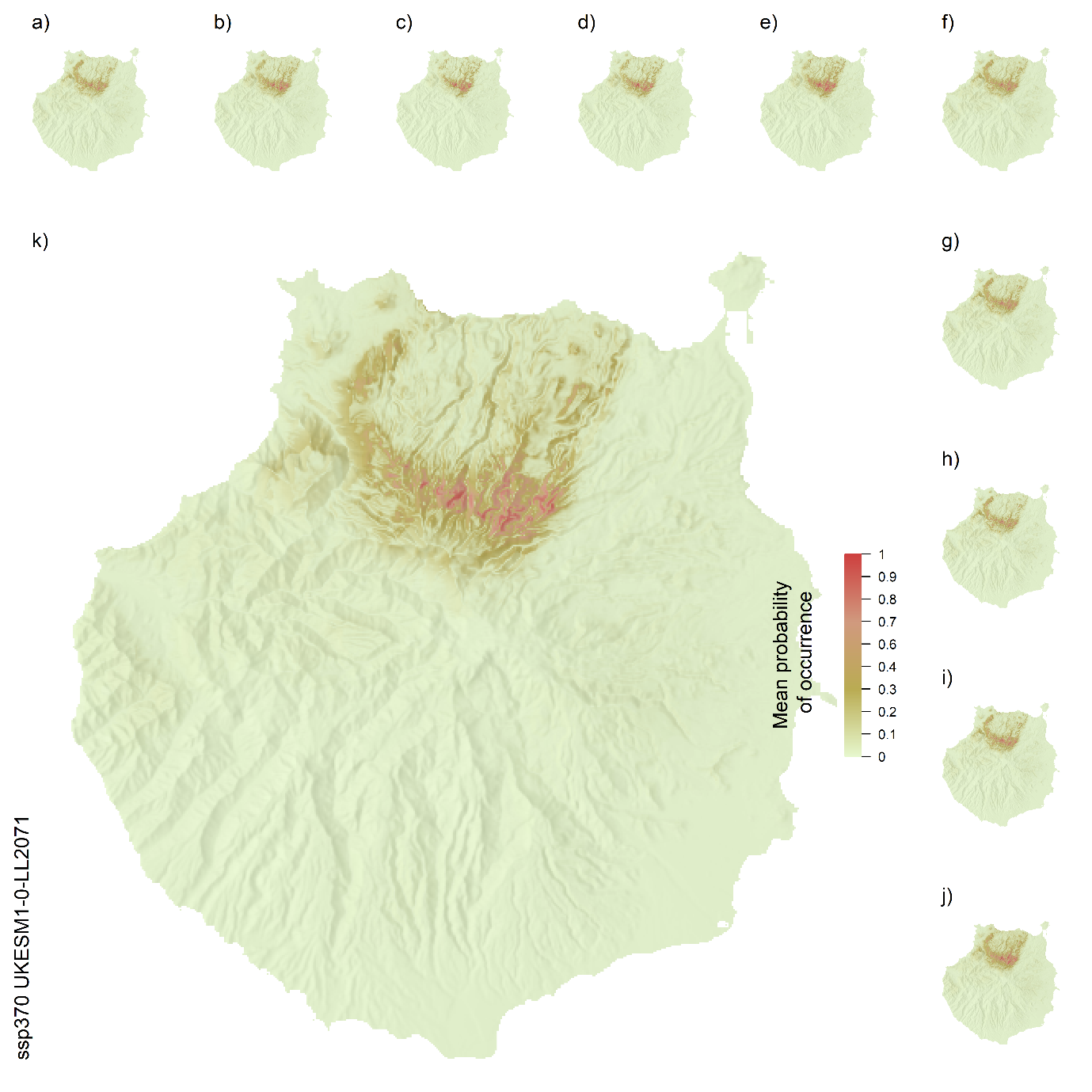
**

**
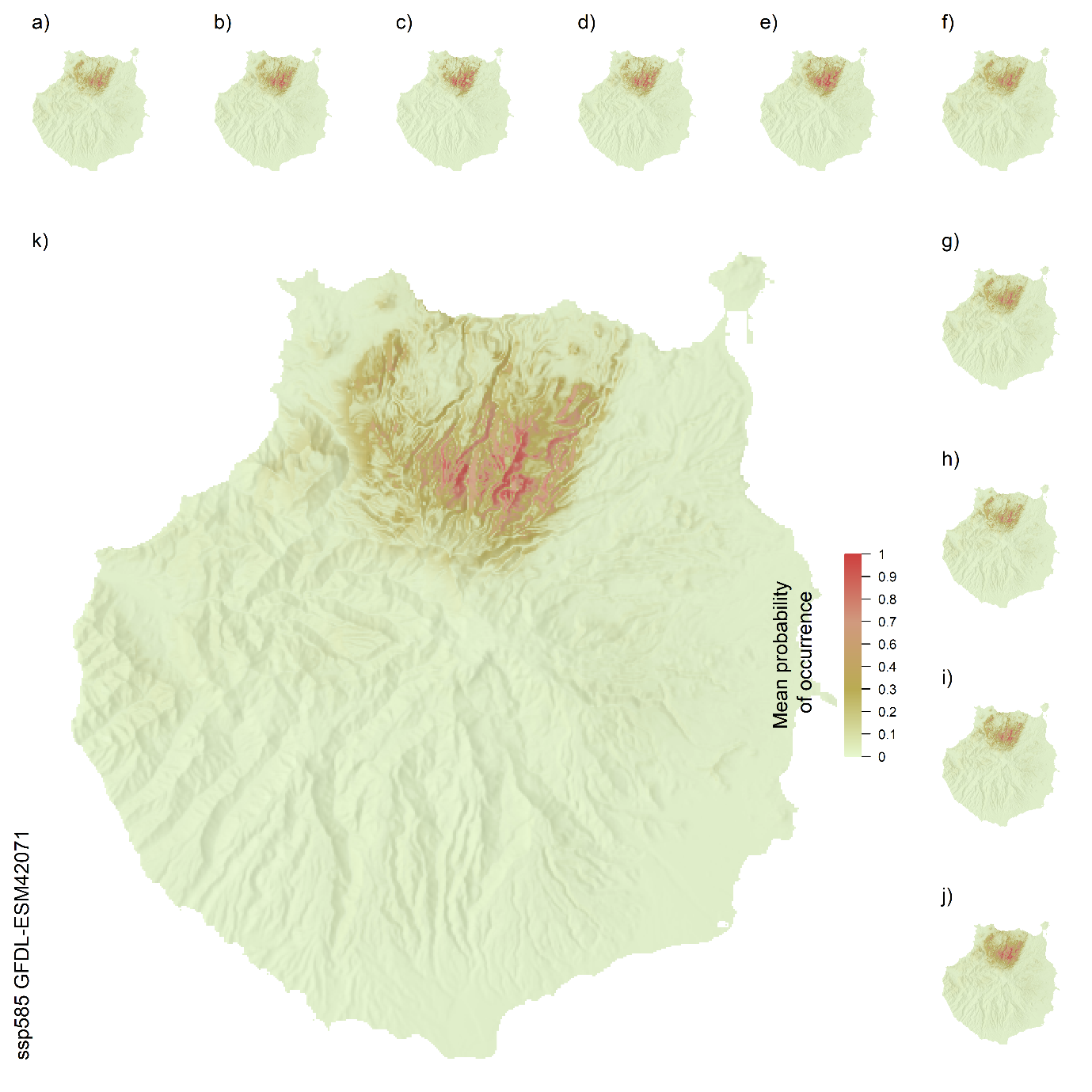

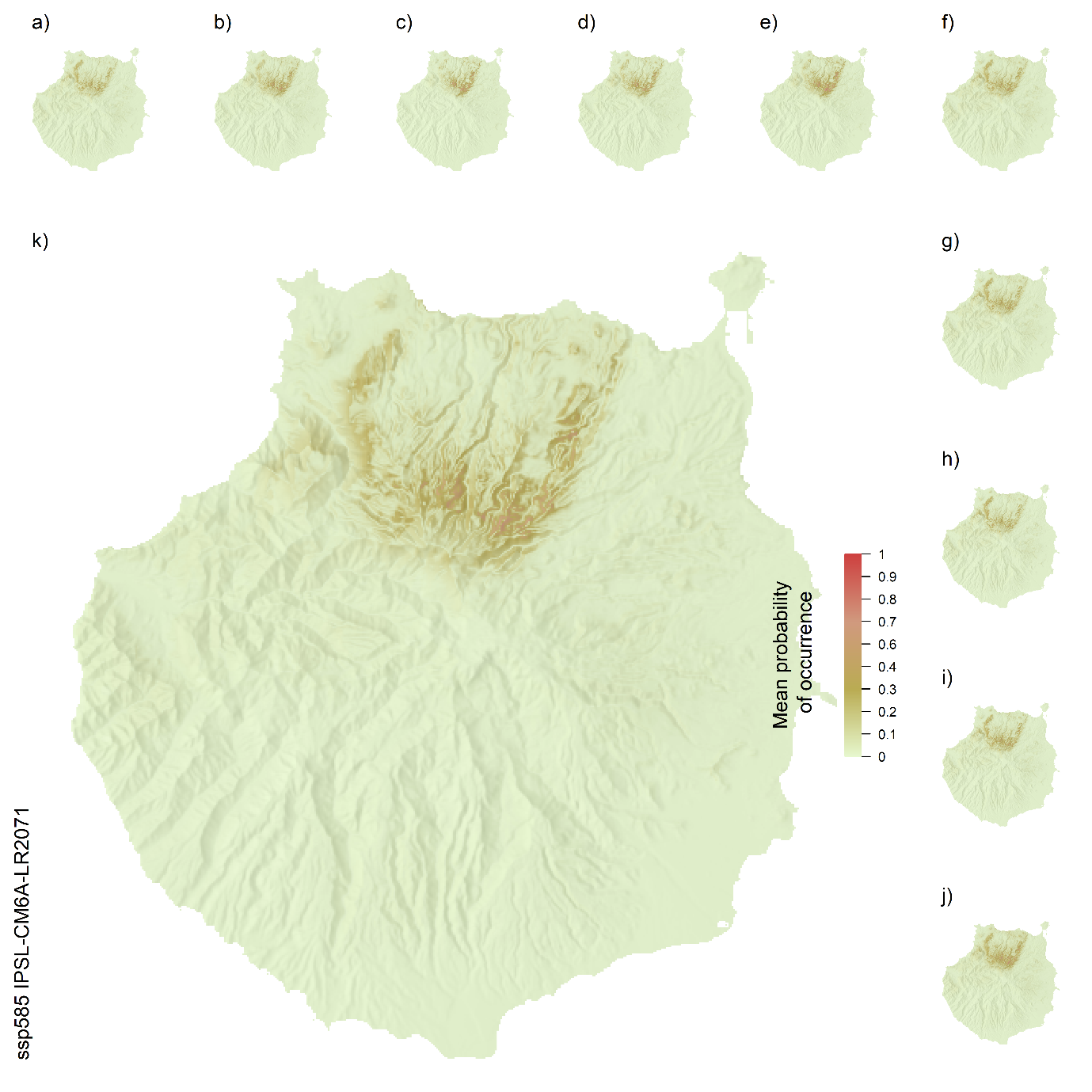

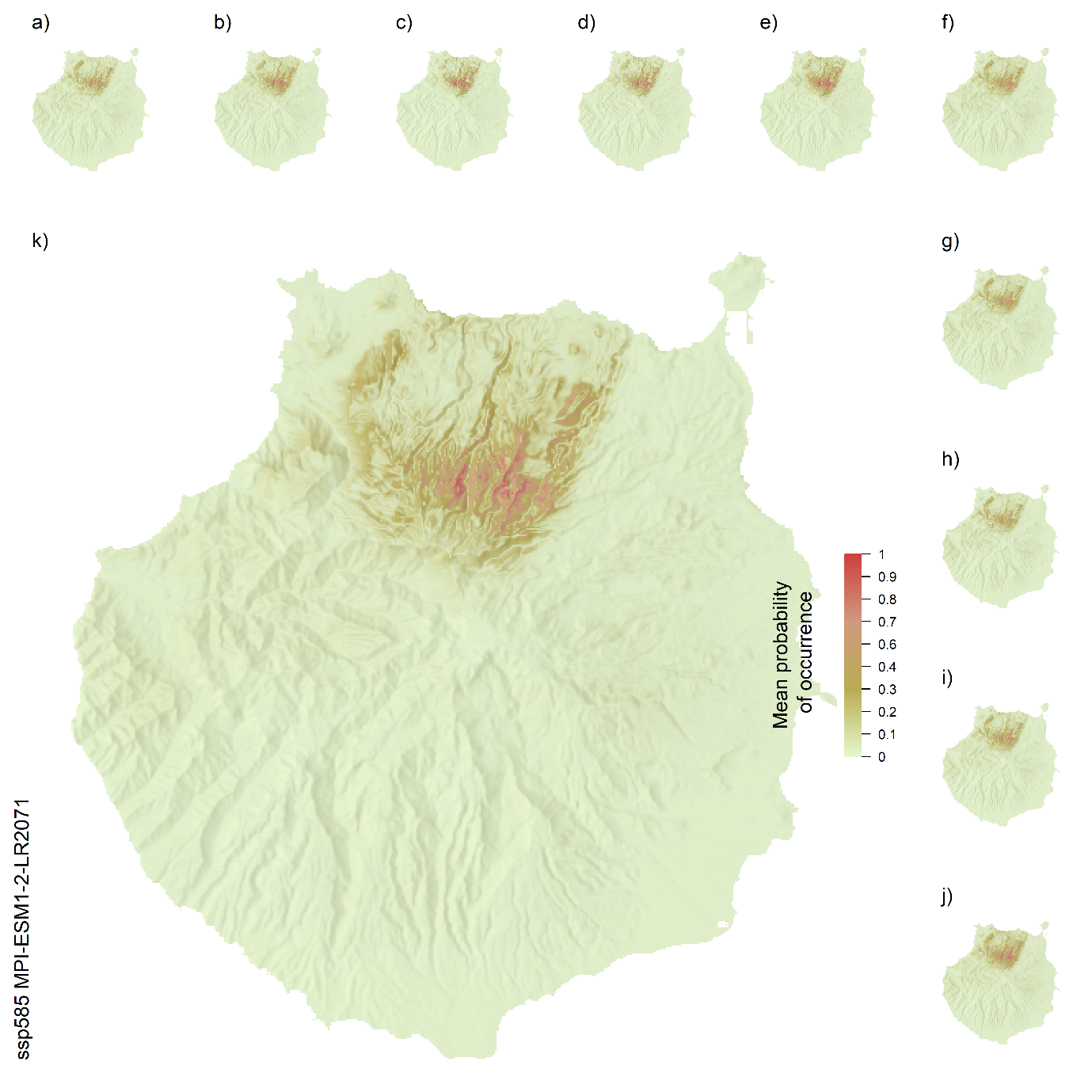

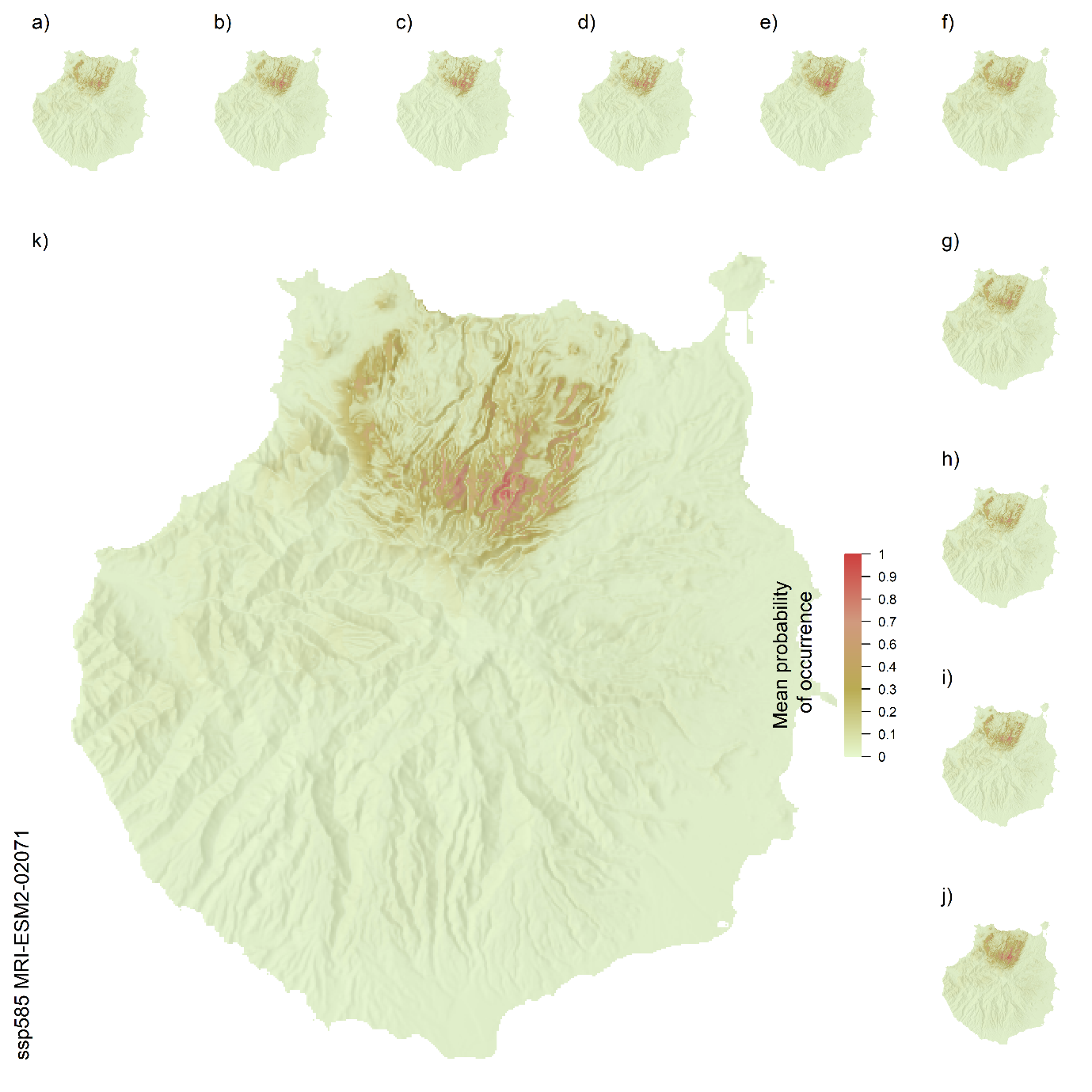

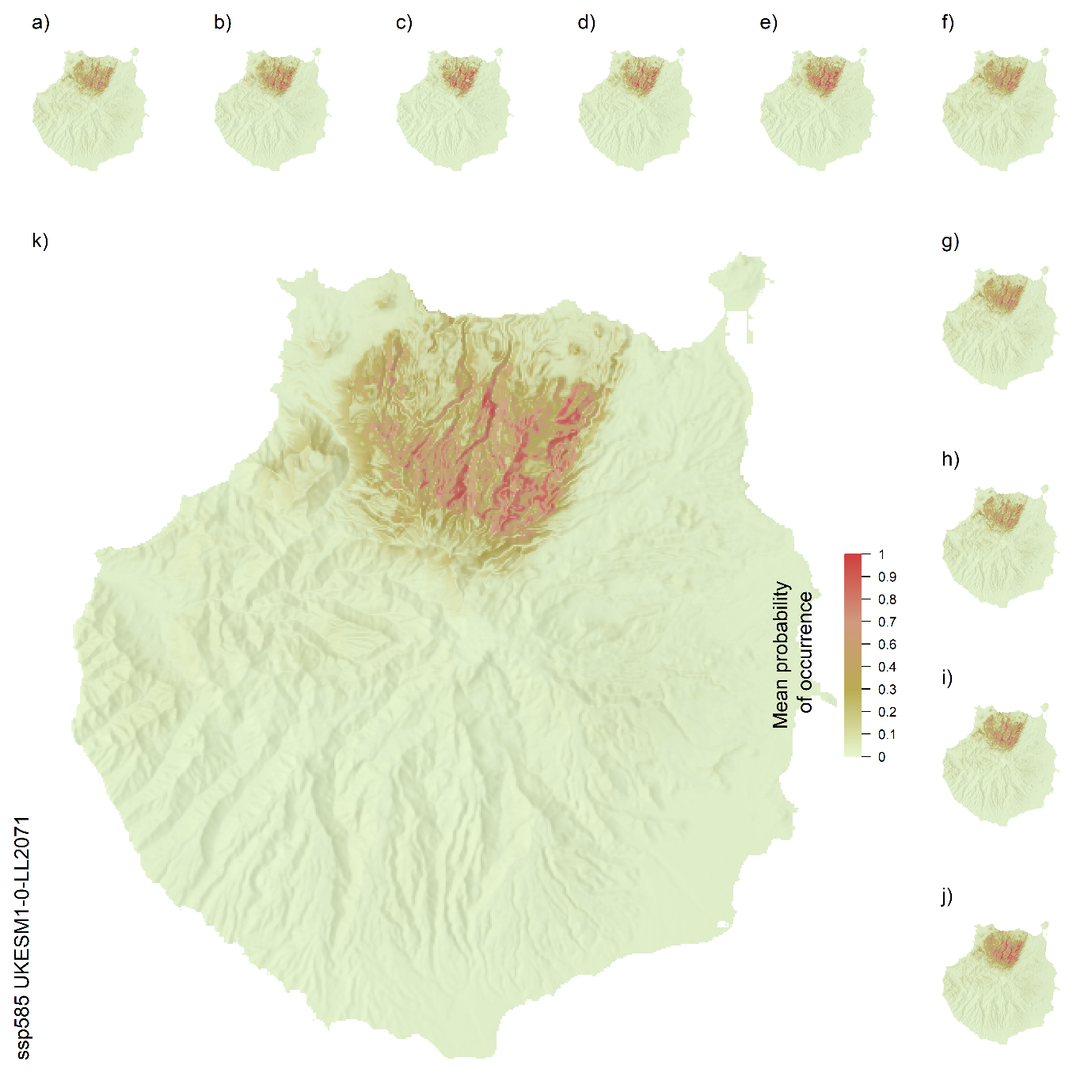
**

**Bibliography**

Albaladejo‐Robles, G., Böhm, M., & Newbold, T. (2022). Species life‐history strategies affect population responses to temperature and land‐cover changes. *Global Change Biology*. https://doi.org/10.1111/gcb.16454

Anderson, R. P. (2013). A framework for using niche models to estimate impacts of climate change on species distributions. *Annals of the New York Academy of Sciences*, *1297*(1), 8–28. https://doi.org/10.1111/nyas.12264

Barbet‐Massin, M., Jiguet, F., Albert, C. H., & Thuiller, W. (2012). Selecting pseudo‐absences for species distribution models: How, where and how many?. *Methods in ecology and evolution*, *3*(2), 327-338.

Beck, J., Böller, M., Erhardt, A., & Schwanghart, W. (2014). Spatial bias in the GBIF database and its effect on modeling species' geographic distributions. *Ecological Informatics*, *19*, 10-15.

Chakraborty, D., Dobor, L., Zolles, A., Hlásny, T., & Schueler, S. (2021). High-resolution gridded climate data for Europe based on bias-corrected EURO-CORDEX: The ECLIPS dataset. *Geoscience Data Journal*, *8*(2), 121–131. https://doi.org/10.1002/gdj3.110

Chamberlain, S., Barve, V., Mcglinn, D., Oldoni, D., Desmet, P., Geffert, L., & Ram, K. (2020). rgbif: Interface to the Global Biodiversity Information Facility API. *R Package Version 2.2.0*. https://cran.r-project.org/package=rgbif.

Fleming, M. D., & Hoffer, R. M. (1979). *Machine Processing of Landsat MSS Data and DMA Topographic Data for Forest Cover Type Mapping*.

Fox, J., & Weisberg, S. (2019). *An R Companion to Applied Regression*. Sage. https://socialsciences.mcmaster.ca/jfox/Books/Companion/

Goslee, S. C., & Urban, D. L. (2007). The ecodist package for dissimilarity-based analysis of ecological data. *Journal of Statistical Software*, *22*(7), 1–19. https://doi.org/10.18637/jss.v022.i07

Guisan, A., Thuiller, W., & Zimmermann, N. E. (2017). Habitat Suitability and Distribution ModelsWith Applications in R. In *Habitat Suitability and Distribution Models: With Applications in R* (pp. i–i). Cambridge University Press.

Hijmans, R. J. (2023). *terra: Spatial Data Analysis*. https://CRAN.R-project.org/package=terra

Latombe, G., Burke, A., Vrac, M., Levavasseur, G., Dumas, C., Kageyama, M., & Ramstein, G. (2018). Comparison of spatial downscaling methods of general circulation model results to study climate variability during the Last Glacial Maximum. *Geoscientific Model Development*, *11*(7), 2563–2579. https://doi.org/10.5194/gmd-11-2563-2018

Li, W., MacBean, N., Ciais, P., Defourny, P., Lamarche, C., Bontemps, S., Houghton, R., & Peng, S. (2017). Gross and net land cover changes based on plant functional types derived from the annual ESA CCI land cover maps. *Earth System Science Data Discussions*, 1–23. https://doi.org/10.5194/essd-2017-74

Miles, J. (2014). Tolerance and Variance Inflation Factor. In *Wiley StatsRef: Statistics Reference Online*. John Wiley & Sons, Ltd. https://doi.org/10.1002/9781118445112.stat06593

Naimi, B., Hamm, N. a s, Groen, T. A., Skidmore, A. K., & Toxopeus, A. G. (2014). Where is positional uncertainty a problem for species distribution modelling. *Ecography*, *37*, 191–203. https://doi.org/10.1111/j.1600-0587.2013.00205.x

O’brien, R. M. (2007). A Caution Regarding Rules of Thumb for Variance Inflation Factors. *Quality & Quantity*, *41*(5), 673–690. https://doi.org/10.1007/s11135-006-9018-6

Patiño, J., Collart, F., Vanderpoorten, A., Martin-Esquivel, J. L., Naranjo-Cigala, A., Mirolo, S., & Karger, D. N. (2023). Spatial resolution impacts projected plant responses to climate change on topographically complex islands. *Diversity and Distributions*, *n/a*(n/a). https://doi.org/10.1111/ddi.13757

Pebesma, E. (2018). Simple Features for R: Standardized Support for Spatial Vector Data. *The R Journal*, *10*(1), 439–446. https://doi.org/10.32614/RJ-2018-009

Phillips, S. J., Anderson, R. P., & Schapire, R. E. (2006). Maximum entropy modeling of species geographic distributions. *Ecological Modelling*, *190*(3–4), 231–259. https://doi.org/10.1016/j.ecolmodel.2005.03.026

Pradhan, P. (2016). Strengthening MaxEnt modelling through screening of redundant explanatory bioclimatic variables with variance inflation factor analysis. *Researcher*, *8*(5), 29–34.

Quinn, G. P., & Keough, M. J. (2002). *Experimental Design and Data Analysis for Biologists*. Cambridge University Press. <https://doi.org/10.1017/CBO9780511806384>

Ritter, P. (1987). *A Vector-Based Slope and Aspect Generation Algorithm*.

Syfert, M. M., Smith, M. J., & Coomes, D. A. (2013). The Effects of Sampling Bias and Model Complexity on the Predictive Performance of MaxEnt Species Distribution Models. *PLOS ONE*, *8*(2), e55158. https://doi.org/10.1371/journal.pone.0055158

Veech, J. A., Small, M. F., & Baccus, J. T. (2011). The effect of habitat on the range expansion of a native and an introduced bird species. *Journal of Biogeography*, *38*(1), 69-77.

Venables, W. N., & Ripley, B. D. (2002). *Modern Applied Statistics with S* (Fourth). Springer. https://www.stats.ox.ac.uk/pub/MASS4/

Wilson, M. F. J., O’Connell, B., Brown, C., Guinan, J. C., & Grehan, A. J. (2007). Multiscale Terrain Analysis of Multibeam Bathymetry Data for Habitat Mapping on the Continental Slope. *Marine Geodesy*, *30*(1–2), 3–35. https://doi.org/10.1080/01490410701295962

Zizka, A., Silvestro, D., Andermann, T., Azevedo, J., Duarte Ritter, C., Edler, D., Farooq, H., Herdean, A., Ariza, M., Scharn, R., Svantesson, S., Wengström, N., Zizka, V., & Antonelli, A. (2019). CoordinateCleaner: Standardized cleaning of occurrence records from biological collection databases. *Methods in Ecology and Evolution*, *10*(5), 744–751. <https://doi.org/10.1111/2041-210X.13152>
